# History for some or lesson for all? A systematic review and meta-analysis on the immediate and long-term mental health impact of the 2002–2003 Severe Acute Respiratory Syndrome (SARS) outbreak

**DOI:** 10.1186/s12889-021-10701-3

**Published:** 2021-04-07

**Authors:** Steven W. H. Chau, Oscar W. H. Wong, Rema Ramakrishnan, Sandra S. M. Chan, Evelyn K. Y. Wong, Pinky Y. T. Li, Vanessa Raymont, Kathryn Elliot, Shanaya Rathod, Gayathri Delanerolle, Peter Phiri

**Affiliations:** 1 Department of Psychiatry, The Chinese University of Hong Kong, Shatin, Hong Kong; 2 Li Chiu Kong Family Sleep Assessment Unit, Department of Psychiatry, Faculty of Medicine, The Chinese University of Hong Kong, Shatin, Hong Kong; 3 Nuffield Department of Women’s and Reproductive Health, University of Oxford, Oxford, UK; 4 Department of Psychiatry, North District Hospital, Sheung Shui, Hong Kong; 5 Oxford Brain Health Clinical Trials Unit, Department of Psychiatry, University of Oxford, Oxford, UK; 6 Research & Development Department, Tom Rudd Unit, Clinical Trials Facility, Moorgreen Hospital, Southern Health NHS Foundation Trust, Southampton, SO30 3JB UK; 7 School of Primary Care, Population Sciences and Medical Education, Faculty of Medicine, University of Southampton, Southampton, UK

**Keywords:** SARS, Infectious disease, Mental health, Post-traumatic stress disorder, Healthcare workers, Covid-19

## Abstract

**Background:**

The aims of this systematic review and meta-analysis are to examine the prevalence of adverse mental health outcomes, both short-term and long-term, among SARS patients, healthcare workers and the general public of SARS-affected regions, and to examine the protective and risk factors associated with these mental health outcomes.

**Methods:**

We conducted a systematic search of the literature using databases such as Medline, Pubmed, Embase, PsycInfo, Web of Science Core Collection, CNKI, the National Central Library Online Catalog and dissertation databases to identify studies in the English or Chinese language published between January 2003 to May 2020 which reported psychological distress and mental health morbidities among SARS patients, healthcare workers, and the general public in regions with major SARS outbreaks.

**Results:**

The literature search yielded 6984 titles. Screening resulted in 80 papers for the review, 35 of which were included in the meta-analysis. The prevalence of post-recovery probable or clinician-diagnosed anxiety disorder, depressive disorder, and post-traumatic stress disorder (PTSD) among SARS survivors were 19, 20 and 28%, respectively. The prevalence of these outcomes among studies conducted within and beyond 6 months post-discharge was not significantly different. Certain aspects of mental health-related quality of life measures among SARS survivors remained impaired beyond 6 months post-discharge. The prevalence of probable depressive disorder and PTSD among healthcare workers post-SARS were 12 and 11%, respectively. The general public had increased anxiety levels during SARS, but whether there was a clinically significant population-wide mental health impact remained inconclusive. Narrative synthesis revealed occupational exposure to SARS patients and perceived stigmatisation to be risk factors for adverse mental health outcomes among healthcare workers, although causality could not be determined due to the limitations of the studies.

**Conclusions:**

The chronicity of psychiatric morbidities among SARS survivors should alert us to the potential long-term mental health complications of covid-19 patients. Healthcare workers working in high-risk venues should be given adequate mental health support. Stigmatisation against patients and healthcare workers should be explored and addressed. The significant risk of bias and high degree of heterogeneity among included studies limited the certainty of the body of evidence of the review.

**Supplementary Information:**

The online version contains supplementary material available at 10.1186/s12889-021-10701-3.

## Background

SARS-CoV-2 has taken the world by storm in a matter of months. While the virus is undoubtedly highly transmissible, another major contributing factor to the catastrophic development of the resulting covid-19 pandemic was the lack of preparedness to battle such a disease. While efforts to contain the virus continue, the collateral damage of this battle on other health-related measures cannot be overlooked. The direct impact of covid-19, as well as social distancing and quarantine measures imposed as a response to the disease, has resulted in the widespread concern that a concurrent mental health crisis is inevitable [1]. Emerging data have shown that the immediate mental health impact on patients and healthcare workers is indeed significant [2–4]. The latest data from Wuhan, the first epicentre of the outbreak, shows that up to one-fourth of patients suffered from sleeping difficulties, anxiety or depression 6 months after being infected with covid-19 [5]. Preparedness for the likelihood of a massive increase in the long-term global mental health burden therefore requires urgent attention.

The Severe Acute Respiratory Syndrome (SARS) outbreak in 2002–2003, while being much more geographically confined and resulting in far less fatalities [6], shares important features with covid-19: (i) both were caused by a novel, contagious and lethal coronavirus capable of causing severe respiratory distress; (ii) both resulted in widespread nosocomial transmission and substantial morbidities among healthcare workers even in the most advanced healthcare systems; and (iii) affected regions imposed rigorous population-wide restrictive measures as a response to contain both viruses [7, 8]. Knowledge about the mental health impact of SARS may thus provide valuable insight into what we can expect in the aftermath of covid-19, which is vitally important for preparedness planning prior to the emergence of long-term data from covid-19 itself.

The key research questions of the review are twofold. First, among SARS patients, healthcare workers, and in the general public in regions of outbreak, what was the prevalence of adverse mental health outcomes during and after the epidemic? Second, what were the protective and risk factors associated with the adverse mental health outcomes in these populations? Provided that the number of studies was sufficient for meta-analysis, we aimed to examine whether the prevalence of adverse mental health outcomes among the target populations changes over time with respect to the course of the illness (i.e. treatment phase, first 6 months post-discharge or beyond 6 months post-discharge for patients) or phase of the epidemic (ie. during epidemic or post-epidemic for non-infected healthcare workers and public). To our knowledge, no systematic review thus far has answered these questions specifically. Even though there are existing reviews that address parts of these questions, significant knowledge gaps exist. For example, Brook et al.’s thematic analysis on studies related to mental health impact and risk factors among healthcare workers did not conduct quantitative analyses [9]. Some recent meta-analyses mixed studies from SARS with studies from outbreaks of other viral infections (e.g. see Kisley et al., Rogers et al. and Yuen at al [10–12].), even though the validity of this approach has not been established due to the differing clinical and social contexts of the outbreaks. Moreover, there are several key methodological limitations among existing reviews. First, studies reported in the Chinese language have generally been omitted in reviews of related topics, a crucial omission given that much of the SARS data was generated in Chinese jurisdictions. Second, these reviews have not taken into account the effect of time since the outbreak on the change in prevalence of mental health outcomes. Third, the existing reviews that examined risk and protective factors associated with mental health outcomes of SARS did not explicitly address inconsistent findings across studies. Bearing these methodological issues in mind, we aimed to address the research questions with a comprehensive systematic review of available evidence.

## Method

### Protocol and registration

The review protocol was registered with the PROSPERO registry (ID: CRD42020183812).

### Eligibility criteria

This review included original studies written in English or Chinese from January 2003 till May 2020 that reported on psychological distress, psychiatric symptoms or diagnoses, and health-related quality of life (HRQoL) among SARS patients, healthcare workers and the general public in the five key outbreak regions (Mainland China, Hong Kong, Canada, Taiwan and Singapore) using standardised measures or clinical assessments by clinicians. Quantitative studies, including observational studies and interventional studies, were included. To ensure comprehensiveness, studies published in peer-reviewed journals or disseminated via other channels, such as conference proceedings or thesis databases, were included, provided full texts of the articles were available for assessment. Case reports and qualitative studies were excluded.

### Search strategies

Major electronic medical and social science publication databases (Pubmed, Medline, EMBASE, Web of Science Core Collection and PsycInfo) were searched using combined search terms that covered SARS and a wide range of mental health conditions. Dissertation databases were searched for unpublished studies. Databases of publications in the Chinese language (e.g. CNKI and National Central Library Online Catalog) were searched using Chinese search terms (see full search terms in Additional file 1). Duplicates were first removed automatically and then manually. The titles and abstracts were screened independently by two reviewers (SWC and OWW), and any disagreement was settled by consensus. Full text articles were reviewed independently by two reviewers (SWC, EKW or SSC) for their eligibility, and any disagreement was also settled by consensus. We conducted a snowball search by identifying additional potentially relevant studies from citation lists of eligible articles. We also contacted corresponding authors of eligible articles through email and asked for their suggestions as to relevant studies and grey literature. SWC checked for overlapping of reported data during the data extraction process. Decisions to exclude articles during the data extraction process were jointly made by three reviewers (SWC, EKW and OWW).

### Data extraction

Data extraction from each article was performed by SWC and one of the two reviewers (SWC, EKW, OWW) in duplicate using a standardised extraction form. Basic characteristics of the studies and data reporting mental health outcomes of relevance, including any psychiatric diagnoses and well-defined measurements of psychiatric symptoms, psychological distress and HRQoL, as well as selected predictors of these outcomes (defined after pilot exploration of the eligible studies) were extracted. Data from studies containing data of selected predictors that were not included in their analyses or results were still extracted, and basic univariate analysis was applied where appropriate. Interventional studies were treated as cross-sectional studies, from which only their baseline assessment data was extracted.

### Data synthesis and analysis

Based on the availability of studies that could be harmonised, the outcomes we were able to include in the meta-analysis were:

i) Prevalence of anxiety disorder among patients and the public.
ii) Prevalence of depressive disorder among patients and healthcare workers.
iii) Prevalence of PTSD among patients and healthcare workers.
iv) Prevalence of significant general psychological distress among healthcare workers.

The statistics extracted from the studies for meta-analysis were either prevalence or mean (standard deviation (SD)). Estimates for studies that reported median and interquartile range (IQR) were converted to mean and SD [13]. Some studies did not report prevalence rates but reported only mean (SD) or median (IQR). For these studies, we employed Monte Carlo simulations to estimate the proportion of the outcome based on appropriate cut-off points for the tool used to measure the outcome. We assumed normality of the distribution when mean (SD) were reported or the data were symmetrical. We used a random effects model to estimate pooled prevalence rates for the above outcomes. We then used a random effects model to compute pooled estimates of the mean scores of HRQoL (mental health, role emotional and social functioning domains from the 36-item Short Form Survey (SF-36)) among patients.

### Quality of evidence assessment

In assessing the overall certainty of the body of evidence from the review, risk of bias in individual studies, inconsistency across studies and publication bias were considered. Risk of bias in individual included studies was independently evaluated by two reviewers (SWC, EKW or OWW) using the Study Quality Assessment Tools by the National Heart, Lung and Blood Institute [14]. Each study was rated as having poor, fair or good quality, which indicates significant, moderate and low risk of bias at the study level, respectively. For assessment of inconsistency across studies, we used the I^2^ statistic to assess heterogeneity between the studies: values of 25, 50, and 75% were used to categorise the degree of heterogeneity into low, moderate and high, respectively [15]. To investigate sources of heterogeneity, we conducted subgroup and sensitivity analyses for outcomes that had sufficient sample sizes. We conducted subgroup analyses by time (relative to the epidemic or discharge from the hospital). Sensitivity analyses were conducted by age (≤40 years vs > 40 years), study design, and outcome measurement tool. We assessed potential publication bias through visualisation of funnel plots and Egger’s test for outcomes with a sample size ≥10. All analyses were conducted using STATA 14.0.

### Reporting standard

The reporting of this study followed the Preferred Reporting Items for Systematic Reviews and Meta-Analyses (PRISMA) guidelines (see PRISMA checklist in Additional file 4).

## Results

### Overview

**Fig. 1:**
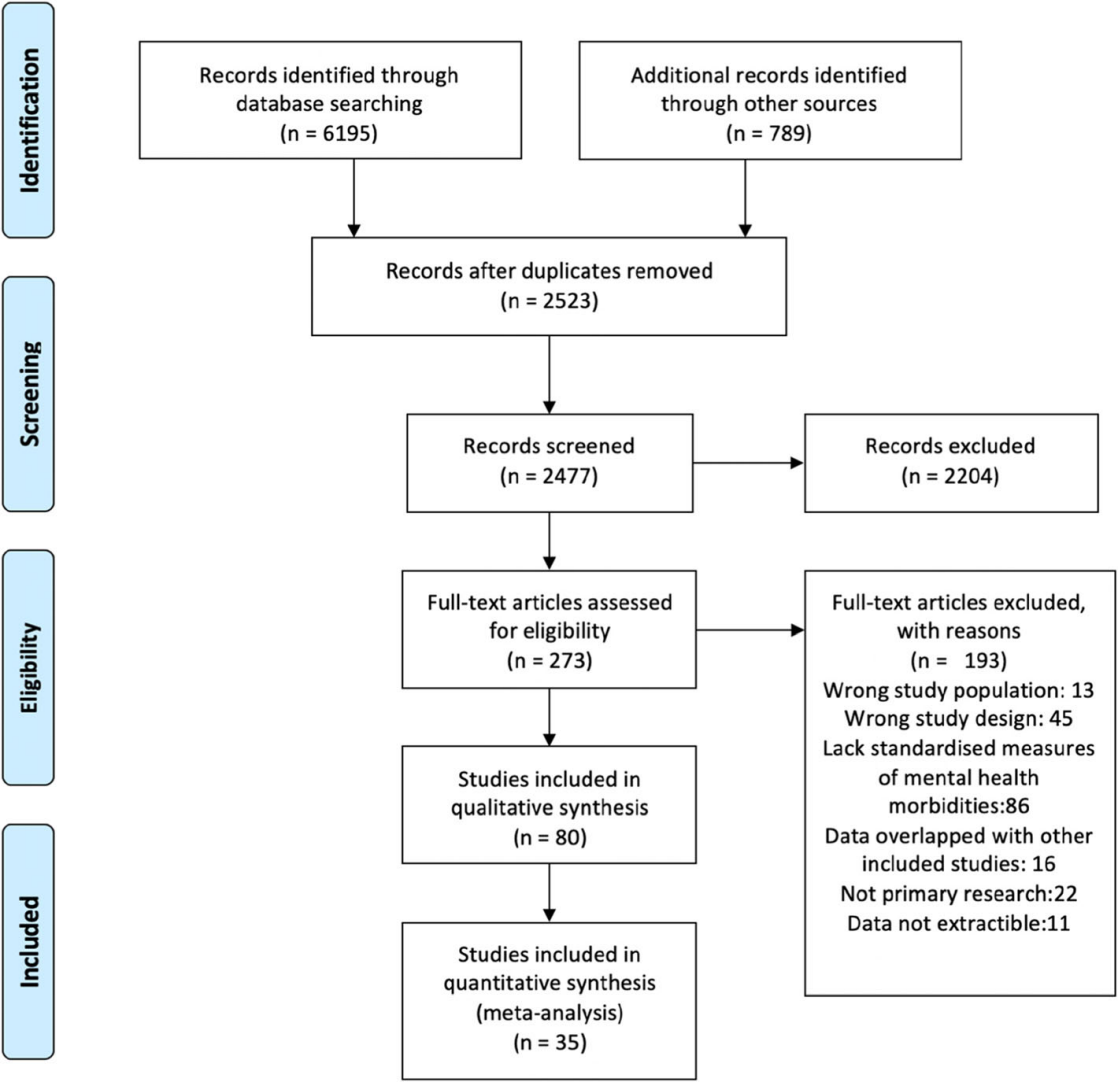
PRISMA flowchart of the review

The major electronic databases yielded 6195 titles. Additional searches from citation lists of included studies and recommendations from authors of included studies yielded an additional 789 titles. After removing 4463 duplicates, 2521 titles and abstracts were screened. Two hundred ninety-four full-text articles were reviewed and eventually 80 studies were included in the systematic review (Fig. 1) (see Additional file 2 for the full list of excluded studies). Thirty-three studies primarily reported mental health outcomes of SARS patients, 28 reported outcomes of healthcare workers of affected regions, and 19 reported outcomes among the general public. Sample sizes ranged from 10 to 10,511. Forty-seven studies were cross-sectional in nature, and only 12 studies used clinical interviews as an assessment tool. Seventeen studies were published in the Chinese language (Table 1A, B & C); and three were unpublished postgraduate theses.

**Table 1:** Complete list of included studies

A.											
	Region of study	Language	Study design	Timeframes of study	Sample size	Age (year)	Female %	HCW%	ICU admission%	Mental health outcomes and measures used	Quality assessment
Liang et al. 2004^#^ [16]	Taiwan	Chinese	Prospective cohort	During treatment, 1 month post discharge	90	55.9	37.8%	20.0%	NR	Anxiety: HADS Depression: HADS PTS: PTSD-RI	**/***
Tang et al. 2005 [17]	Mainland China	Chinese	Retrospective	During treatment	325	38.3	67.1%	NR	NR	Any neuropsychiatric disturbances (including psychosis and delirium): Clinician assessment	*/*
Wang J et al. 2005 [18]	Mainland China	Chinese	Prospective cohort	During treatment (multiple timepoints)	29	37.5	48%	NR	NR	General psychological distress: SCL-90	*/**
Huang et al. 2004 [19]	Mainland China	Chinese	cross-sectional	During treatment	218	81% within 31 to 41	79.8%	50%	NR	Depression: SDS	*/**
Lee D et al. 2004 [20]	Hong Kong	English	Case-control	During treatment	Psychotic cases: 15; Non- psychotic control: 30	32.8	60%	NR	6.7%	Psychosis: Clinician assessment	***/***
Cheng S et al. 2004 (a) [21]	Hong Kong	English	Cross-sectional	During treatment	10	34.8	60%	NR	NR	Any psychiatric disorder: Clinician assessment	**/**
Chua et al. 2004(a) [22]	Hong Kong	English	Cross-sectional	During treatment	79 (non-patient control:145)	18–60	61.1%	39%	NR	Stress/burnout: PSS-10	**/**
Wang W et al. 2005 [23]	Mainland China	English	Cross-sectional	During treatment	103	82% within 20 to 50	59%	NR	NR	General psychological distress: SCL-90	*/*
Hu et al. 2004 [24]	Mainland China	Chinese	Cross-sectional	During treatment	35 (Non-patient control: 50)	32	42.9%	NR	NR	General psychological distress: SCL-90 Anxiety: SAS Depression: SDS	*/**
Kwek et al. 2006 [25]	Singapore	English	Cross-sectional	3 months post-discharge	63	34.8	79.4%	54%	9.5%	Anxiety: HADS Depression: HADS PTS: IES HRQoL: SF-36	**/***
Hui et al. 2005 [26]	Hong Kong	English	Prospective cohort	3 months, 6 months and 12 months post-onset	97	36.9	66%	NR	32.0%	HRQoL: SF-36	***/***
Cheng S et al. 2004 (b) [27]	Hong Kong	English	Cross-sectional	1–2 months post discharge	180	36.9	66.7%	25%	11.1%	Anxiety: BAI Depression: BDI	**/***
Cheng S et al. 2004(c) [28]	Hong Kong	English	Cross-sectional	1 month post discharge	100 (Non-patient control: 184)	37.1	66%	18%	9%	General psychological distress: GHQ-28 HRQoL: WHOQOL-BREF	*/**
Wu et al. 2005 [29]	Hong Kong	English	Cross-sectional (multiple timepoints)	1 month and 3 month post discharge	131	41.8	56%	11%	11%	Anxiety: HADS Depression: HADS PTS: IES-R	**/**
Xu et al. 2005 [30]	Mainland China	Chinese	Cross-sectional	3 months post discharge	114 (non-patient control: 93)	36.9	54%	NR	NR	PTS: IES-R	*/*
Hong et al. 2009 [31]	Mainland China	English	Prospective cohort	Multiple timepoints from 1 to 46 months post-discharge	70	38.5	67.1%	NR	NR	PTS: Clinician assessment by CCMD III criteria	***/***
Fang et al. 2004 [32]	Mainland China	Chinese	cross-sectional	3 months post discharge	286	33.4	52.8%	21.7%	NR	Anxiety: SAS Depression: SDS PTS: Clinician assessment by DSM4 criteria	**/**
Sun et al. 2005 [33]	Mainland China	Chinese	Prospective cohort	3 months and 12 months post discharge	114	36.9	54%	NR	NR	PTS: Clinician assessment by DSM4 criteria	*/**
Lin et al. 2004 [34]	Mainland China	Chinese	cross-sectional	Early post discharge	45 (Non-patient control: 45)	33.5	52%	NR	NR	General psychological distress: SCL-90	*/**
Lau H et al. 2005 [35]	Hong Kong	English	RCT	2 weeks post discharge	133	37	66.2%	NR	NR	HRQoL: SF-36	*/**
Shi et al. 2011 [36]	Mainland China	Chinese	Prospective cohort	Early post discharge and 5 years post discharge	43 (Non-patient control: 41)	30.6	88%	100%	NR	Depression: SDS Neurocognitive function	*/**
Peng C et al. 2005 [37]	Mainland China	Chinese	cross-sectional	3 months post discharge	117	36.5	53.8%	NR	NR	General psychological distress: SCL-90	*/*
Cheng et al. 2006 [38]	Hong Kong	English	cross-sectional	2 to 6 months post discharge	57	38.1	66.7%	38.6%	17.5%	Anxiety: BAI Depression: BDI	**/***
Tansey et al. 2007 [39]	Canada	English	Prospective cohort	First year post discharge	107	42	67%	65%	16%	HRQoL: SF-36 Psychiatric service utilisation	**/***
Lee A et al. 2007 [40]	Hong Kong	English	cross-sectional	1 year post SARS	96	NR	63.5%	34.4%	NR	Anxiety: DASS Depression: DASS PTS: IES-10 Stress/burnout: PSS-10	**/**
Gao et al. 2006 [41]	Mainland China	Chinese	cross-sectional	1 year post SARS	61	25.3	68.7%	NR	NR	PTS: Clinician assessment by CCMD III criteria	*/**
Moldofsky et al. 2011 [42]	Canada	English	retrospective	1–3 years post SARS	21	46.3	90.5%	90.50%	NR	Depression: BDI PTS: PCL-C Sleep: Polysomnography Fatigue	*/**
Lam et al. 2009 [43]	Hong Kong	English	cross-sectional	3–4 years post SARS	181	44.7	68.5%	47.5%	11.7%	Any psychiatric diagnosis: Clinician assessment by DSM4 criteria Fatigue: CFQ	***/***
Mak et al. 2009, Mak et al. 2010 and Yip 2015# [44–46]	Hong Kong	English	Prospective cohort	2–3 years and 10 years post SARS	90	41.1	62.2%	30%	10%	Any psychiatric diagnosis: Clinician assessment by DSM4, HADS HRQoL: SF-36	***/***
Guo et al. 2019 [47]	Mainland China	English	Prospective cohort	12 years post SARS	67	33	57%	NR	NR	HRQoL: SF-36	**/**
B.											
	Region of study	Language	Study design	Timeframes of study	Specific population		Sample size	Age (year)	Female %	Mental health outcomes and measures used	Quality assessment
Chen CS et al. 2005 [48]	Taiwan	English	Prospective cohort	During epidemic (multiple timepoints)	Nurses of SARS units		116	31	98.3%	Anxiety: SAS Depression: SDS Sleep: PSQI	*/**
Nickell et al. 2004 [49]	Canada	English	Cross-sectional	During epidemic	HCWs of SARS hospital		510	NR	81.1%	General psychological distress: GHQ-12	*/**
Chong et al. 2004 [50]	Taiwan	English	Cross-sectional	During epidemic	HCWs of SARS hospital		1257	31.8	81%	General psychological distress: CHQ PTS: IES	***/***
Xu et al. 2004 [51]	Mainland China	Chinese	Cross-sectional	During epidemic	HCWs of SARS hospital		89	30.6	91%	PTS: IES-R	**/**
Chen N et al. 2007 [52]	Taiwan	English	Prospective cohort	During epidemic	HCWs of SARS hospital		172	31.4	NR	HRQoL: SF-36	**/**
Chen C et al. 2005 [53]	Taiwan	English	Cross-sectional	During epidemic	Nurses of SARS hospital		128	26.5	100%	General psychological distress: SCL-90-R PTS: IES	**/**
Maunder et al. 2004 [54]	Canada	English	cross-sectional	During epidemic	HCWs of SARS outbreak region		1557	40.2	74.6%	PTS: IES	**/**
Zhang et al. 2005 [55]	Mainland China	Chinese	Cross-sectional	During epidemic	HCWs of SARS hospital		HCWs: 94; general public control: 58	31.5	72.3%	General psychological distress: SCL-90	*/*
Su et al. 2007 [56]	Taiwan	English	Prospective cohort	During epidemic (multiple timepoints)	Nurses of SARS hospital		102	25.4	100%	Anxiety: STAI, clinician assessment by M.I.N.I. Depression: BDI, M.I.N.I. PTS: DTS-C, M.I.N.I. Sleep: PSQI	**/**
McAlonan et al. 2007 [57]	Hong Kong	English	cross-sectional (multiple timepoint)	During epidemic and 1 year after	HCWs of SARS hospital		360	30–50	68.3%	Anxiety: DASS-21 Depression: DASS-21 PTS: IES-R Stress/burnout: PSS-10	**/**
Poon et al. 2004 [58]	Hong Kong	English	cross-sectional	During epidemic	HCWs of SARS hospital		1926	NR	NR	Anxiety: STAI Stress/burnout: MBI	**/**
Chua et al. 2004(b) [59]	Hong Kong	English	cross-sectional	During epidemic	HCWs of SARS hospital		HCWs:271; general public control: 342	Most within 19–40	75%	Stress/burnout: PSS-10	**/**
Fiksenbaum et al. 2006 [60]	Canada	English	cross-sectional	During epidemic	Nurses of SARS hospital		333	43.8	93.6%	Stress/burnout: MBI	*/*
Wong S 2004# [61]	Hong Kong	English	Prospective cohort (only baseline data contains measures of interest)	During epidemic	HCWs of SARS hospital		87	NR	57.5%	PTS: IES	*/*
Tam et al. 2004 [62]	Hong Kong	English	cross-sectional	During epidemic	HCWs of SARS hospital		652	34.1	79%	General psychological distress: GHQ-12	**/**
Verma et al. 2004 [63]	Singapore	English	cross-sectional	During epidemic	General practitioners and traditional Chinese medicine practitioners		1050	46.6	39.7%	General psychological distress: GHQ-28 PTS: IES-R	**/**
Koh et al. 2005 [64]	Singapore	English	cross-sectional	During epidemic	HCWs of SARS outbreak region		10,511	36.6	82%	PTS: IES	**/**
Sim et al. 2004 [65]	Singapore	English	cross-sectional	During epidemic	HCWs of SARS hospital		47	Majority within 25–50	29.8%	General psychological distress: GHQ-28 PTS: IES	**/**
Styra et al. 2008 [66]	Canada	English	cross-sectional	1–2 months post- epidemic	HCWs of SARS hospital		248	36.9	86%	PTS: IES-R	***/***
Tham et al. 2004 [67]	Singapore	English	cross-sectional	6 months post-epidemic	HCWs of SARS hospital		96	31.9	68.8%	General psychological distress: GHQ-28 PTS: IES	**/***
Lung et al. 2009 [68]	Taiwan	English	Prospective cohort	1–9 months post epidemic, 1 year post epidemic	HCWs with direct clinical contact with SARS patients		127	32.3	58%	General psychological distress: CHQ-12	**/**
Lin et al. 2007 [69]	Taiwan	English	cross-sectional	1 month post epidemic	HCWs of SARS hospital		92	33.8	91.3%	General psychological distress: CHQ-12 PTS: DTS-C	**/**
Chen CC et al. 2004 [70]	Taiwan	Chinese	cross-sectional	1 month post epidemic	HCWs of SARS outbreak region		222	36	94%	General psychological distress: BSRS	*/**
Maunder et al. 2006 & Lancee et al. 2008 [71, 72]	Canada	English	cross-sectional	1–2 years post epidemic	HCWs of SARS outbreak region		769 (a subset of 139 for Lancee et al.)	NR	86.9%	General psychological distress: K10 PTS: IES Any clinical psychiatric diagnoses: Clinician assessment by DSM4 Stress/burnout: MBI-EE	Maunder et al.: **/**; Lancee et al.: */**
Wu et al. 2008, 2009 & Liu at al. 2127 [73–75]	Mainland China	English	cross-sectional	3 years post epidemic	HCWs of SARS hospital		539	47.1% within 36–50	76.5%	Depression: CES-D PTS: IES-R Alcohol use	**/** (all three)
C.											
	Region of study	Language	Study design	Timeframes of study	Specific population		Sample size	Age (year)	Female %	Mental health outcomes and measures used	Quality assessment
Yu et al. 2005 [76]	Hong Kong	English	Prospective cohort	Pre-SARS, during epidemic	Middle age women		126	56.7	100%	Depression: CES-D Stress/burnout: PSS-10	*/**
Leung et al. 2005 [77]	Hong Kong	English	Cross-sectional (multiple timepoints)	During epidemic (multiple timepoints), 6 months post SARS	General public		4481	NR	57%	Anxiety: STAI-state (10 items)	***/***
Dang et al. 2004 [78]	Mainland China	Chinese	Cross-sectional	During epidemic	College students		6280	NR	39.4%	Anxiety: SAS Depression: SDS	*/**
Cheng C et al. 2005 [53]	Hong Kong	English	Prospective cohort	During epidemic (multiple timepoints)	College students		72	21.1	57%	Anxiety: STAI	**/***
Wong T et al. 2007 [79]	Hong Kong	English	Cross-sectional	During epidemic	College students		763	22.2	31.6%	Anxiety: SAS	*/*
Lee D et al. 2006 [80]	Hong Kong	English	Cross-sectional	During epidemic (with pre-SARS data as reference)	Pregnant women		235	29.6	100%	Anxiety: STAI Depression: BDI	**/***
Chang et al. 2004 [81]	Singapore	English	Cross-sectional	During epidemic	General public		174	31.0	55%	Anxiety: STAI Depression: CES-D	*/*
Chan et al. 2007 [82]	Hong Kong	English	Non-controlled interventional	During epidemic	Senior citizens		122	NR	63%	Anxiety: STAI	*/**
Jin et al. 2003 [83]	Mainland China	Chinese	Non-controlled interventional	During epidemic	College students		138	18–23	NR	Anxiety: SAS	*/*
Wang JD 2003 [84]	Mainland China	Chinese	Cross-sectional	During epidemic	Non-SARS patients to a hospital		114	16–60	35.7%	Anxiety: SAS Depression: SDS General psychological distress: SCL-90	*/*
Lau J et al. 2006 [85]	Hong Kong	English	Cross-sectional	During epidemic	General public		818	79% within 18–49	48.8%	PTS: IES	**/**
Hawryluck et al. 2004 [86]	Canada	English	Cross-sectional	During epidemic	Quarantined general public		129	80% within 26–55	NR	Depression: CES-D PTS: IES-R	**/**
Reynolds et al. 2008 [87]	Canada	English	Cross-sectional	Immediate post- epidemic	Quarantined general public		1057	51.9	55.5%	PTS: IES-R	**/***
Peng E et al. 2010 [37]	Taiwan	English	Cross-sectional	4–5 months post epidemic	General public		1278	41.6	49.7%	General psychological distress: BSRS-5	**/***
Ko et al. 2006 [88]	Taiwan	English	Cross-sectional	1 month post epidemic	General public		1449	76% within 15–50	49.3%	Depression: TDQ	**/**
Ng et al. 2006 [89]	Hong Kong	English	RCT	2 months post epidemic	General public with chronic illness		34	55.3	74.5	Anxiety: BSI Depression: BSI	*/*
Lee T et al. 2006 [80]	Hong Kong	English	Cross-sectional	3 months post epidemic	General public		146	61.1	NR	Depression: CES-D PTS: IES-R	**/**
Cheung et al. 2008 [90]	Hong Kong	English	Retrospective	The year of SARS	Senior citizens		/	NR	NR	Suicide: Cause of death from death registry	**/***
Tan 2019 [91]	Taiwan	English	Retrospective	The year of SARS	General public		/	NR	NR	Depression: Diagnosis from health insurance registry	*/*

List of included studies of which A. SARS patients, B. non-infected healthcare workers of SARS affected regions and C. general public of SARS affected regions were the primary study population, respectively

*SARS* severe acute respiratory syndrome, *HCWs* healthcare workers, *NR* not reported, *PTS* post-traumatic stress, *BAI* Beck anxiety inventory, *BDI* Beck depression inventory, *BSRS* Brief symptoms rating scale, *CCMD-III* Chinese classification of mental disorders 3rd version, *CES-D* Center for Epidemiologic Studies Depression Scale, *CHQ-12* Chinese Health Questionnaire, *DSM4* Diagnostic and statistical manual of mental disorders 4th version, *DASS* Depression Anxiety Stress Scales, *DTS-C* Davidson Trauma Scale Chinese version, *GHQ-12* General Health Questionnaire-12 items, *GHQ-28* General Health Questionnaire-28 items, *HADS* Hospital anxiety and depression scale, *ICD-10* International classification of disease 10th version, *IES* Impact of event scale, *IES-R* Impact of event scale-revised, *K-10* Kessler Psychological Distress Scale, *MBI* Maslach Burnout Inventory, *M.I.N.I.* The Mini-International Neuropsychiatric Interview, *PCL-C* PTSD Checklist Chinese version, *PSQI* Pittsburgh Sleep Quality Index, *PSS* Perceived stress scale, *PTSD-RI* Post-Traumatic Stress Disorder reaction index, *SAS* Zung’s self-rating anxiety scale, *SDS* Zung’s self-rating depression scale, *SCL-90* Symptoms checklist-90, *SCL-90-R* Symptoms checklist-90 revised, *SF-36* The 36-Item Short Form Survey, *STAI* State-trait anxiety scale, *TDQ* Taiwanese Depression Questionnaire

# unpublished theses

*/**/*** refer to poor/fair/good quality as rated by two reviewers independently using the Study Quality Assessment Tools by the National Heart, Lung and Blood Institute

### SARS patients

#### Treatment phase

Nine studies described psychiatric morbidities during treatment phase [16–24]. As expected, studies generally reported a high level of psychological distress among SARS patients during the acute phase, as compared to the general population or patients of other illnesses (except for Wang et al. [23]). Psychotic disorders were also reported - one study from Hong Kong reported a prevalence of 0.9% [20]. The aetiology of acute phase psychotic disorder has generally been assumed to be due to the ultra-high dose of steroid treatment, because such occurrence has been reported to be associated with higher steroid dosage, and symptoms have resolved when steroid dosage was reduced [20, 21]. One Chinese study reported post-mortem findings of perivascular mononuclear cell and lymphocyte infiltration and neuronal demyelination in two patients with psychosis [17], although there was no description of the clinical details of these cases and it is thus difficult to establish any relationship between these findings and psychosis. The same study also reported high mortality rates associated with psychotic disorder (61.5% among those with psychosis or delirium). Lee et al. [20] found that a family history of mental illness was significantly associated with psychotic disorder during treatment, suggesting that an underlying personal or biological vulnerability could have contributed to this phenomenon. However, since the results of this study were based on retrospective analysis of case records, the family history of mental illness of those who were not psychotic could have been neglected.

#### Post-recovery period

Twenty-four studies reported post-recovery mental health outcomes [16, 25–46], which covered the timeframe of 2 weeks to 12 years post-discharge. The most commonly reported outcomes were PTSD, depression, anxiety and HRQoL.

**Fig. 2:**
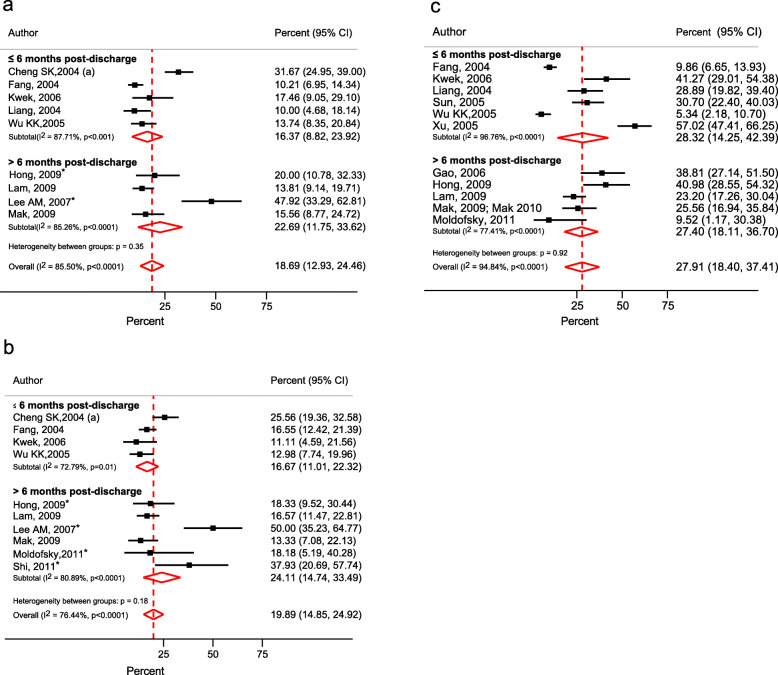
Forest plots of pooled estimates of prevalence of mental health outcomes among SARS survivors. **a** Forest plot of pooled estimate of prevalence of probable or clinically diagnosed anxiety disorder among SARS patients in post-recovery period; **b** Forest plot of pooled estimate of prevalence of probable or clinically diagnosed depressive disorder among SARS patients in post-recovery period; **c** Forest plot of pooled estimate of prevalence of post-traumatic stress disorder (PTSD) among SARS patients in post-recovery period. Note: *Prevalence calculated from mean and standard deviation using Monte Carlo simulation

The point prevalence of probable or clinician-diagnosed anxiety disorders among SARS patients after discharge was 18.7% (95% CI: 12.9–24.5%, I^2^ = 85.5%; from nine studies [16, 25, 28, 29, 32, 40] with total *n* = 1127). Using a cut-off of 6 months, the point prevalence of anxiety disorders among this group during the early post-discharge period was 16.4% (95% CI: 8.8–23.9%, I^2^ = 87.7%; from five studies with total *n* = 748), with 22.7% beyond that period (95% CI: 11.8–33.6%, I^2^ = 85.3%; from four studies with total *n* = 379). The point prevalence of the two time periods were not significantly different (*p* = 0.35) (Fig. 2a).

The point prevalence of probable or clinician-diagnosed depressive disorder among SARS patients after discharge was 19.9% (95% CI: 14.9–24.9%, I^2^ = 76.4%; from 10 studies [25, 28, 29, 31, 32, 36, 40, 42–44] with total *n* = 1088). Using a cut-off of 6 months, the point prevalence of depression among this group during the early post-discharge period was 16.7% (95% CI: 11.0–22.3%, I^2^ = 72.8%; from four studies with total *n* = 658), with 24.1% beyond that period (95% CI: 14.7–33.5%, I^2^ = 80.9%; from six studies with total *n* = 430). The point prevalence of the two time periods were not significantly different (*p* = 0.18) (Fig. 2b).

The point prevalence of probable or clinician-diagnosed PTSD among SARS patients after discharge was 27.9% (95% CI: 18.4–37.4%, I^2^ = 94.8%; from 11 studies [16, 30–33, 41–45] with total *n* = 1216). Using a cut-off of 6 months, the point prevalence of PTSD among this group during the early post-discharge period was 28.3% (95% CI: 14.3–42.4%, I^2^ = 96.8%; from six studies with total *n* = 796), with 27.4% beyond that period (95% CI: 18.1–36.7%, I^2^ = 77.4%; from five studies with total *n* = 420) (Fig. 2c).

For post-discharge HRQoL, five studies that used the SF-36 measures were included in the meta-analysis. We focused on the domains that are relevant to mental health, namely the mental health, role-emotional, and social functioning domains. The pooled estimates of the mean of the domains were as follows: mental health: 66.6 (95% CI: 63.3–69.8, I^2^ = 51.3%; from five studies [26, 31, 35, 44, 47] with total *n* = 277); role emotional: 57.4 (95% CI: 48.1–66.6, I^2^ = 68.8%; from five studies [26, 31, 35, 44, 47] with total *n* = 277); and social functioning: 70.5 (95% CI: 61.4–79.7, I^2^ = 88.4%; from five studies [26, 31, 35, 44, 47] with total *n* = 277) (see Supplementary Figures 1–3).

**Fig. 3:**
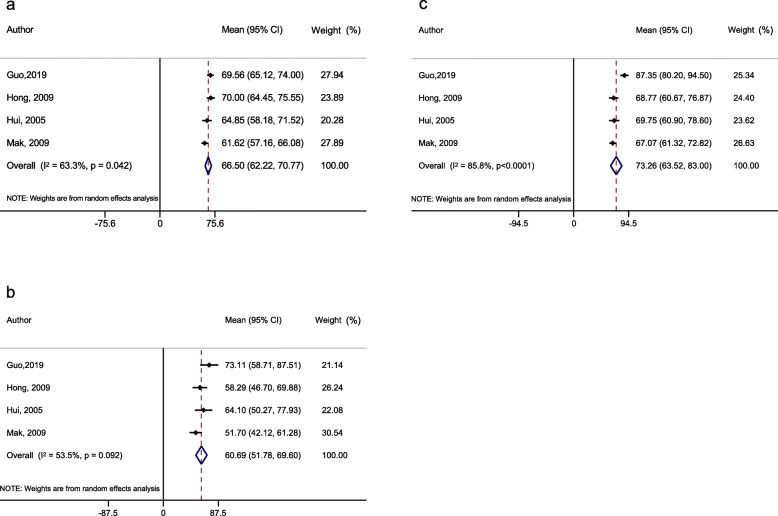
Forest plots of pooled estimates of health-related quality of life measures of SARS survivors. **a** Forest plot of pooled estimate of mean of 36-item Short Form Survey (SF-36) mental health domain among SARS patients in > 6 months post-discharge; **b** Forest plot of pooled estimate of mean of SF-36 role emotional domain among SARS patients in > 6 months post-discharge; **c** Forest plot of pooled estimate of mean of SF-36 role social functioning among SARS patients in > 6 months post-discharge

Subgroup analysis of studies conducted beyond 6 months post-discharge yielded the following pooled estimates of mean score: mental health: 66.5 (95% CI: 51.8–69.6, I^2^ = 63.3%; from four studies with total *n* = 206); role emotional: 60.7 (95% CI: 62.2–70.8, I^2^ = 53.5%; from four studies with total *n* = 206); and social functioning: 73.3 (95% CI: 63.5–83, I^2^ = 85.8%; from four studies with total *n* = 206) (Fig. 3a, b & c). Since these studies were all conducted on Chinese populations, we referred to the SF-36 norm of Hong Kong Chinese for comparison [92] (mental health: 72.8, SD 16.6; role emotional: 71.7, SD 38.4; and social functioning: 91.2, SD 16.5). The comparison showed that the pooled mean scores of the three domains were below population norm. In particular, social functioning was more than 1 SD below norm. Only one study reported HRQoL measures within 6 months post-discharge, and thus we could not compare the estimates for before and beyond 6 months post-discharge.

Two studies reported chronic fatigue symptoms among SARS survivors beyond the first year of recovery. Lam et al. reported that up to 40% of the cohort fulfilled the criteria for chronic fatigue syndrome (CFS) [92]. Among those who fulfilled the criteria for CFS, approximately half were without a psychiatric diagnosis. Moldofsky and Patcal reported excessive post-sleep fatigue among 21 survivors, with significant persistent functional impairment, together with diffuse myalgia and subjective weakness [42]. Polysomnography showed that the subjects had sleep instability, frequent arousal and an increase in REM onset latency, with fatigue and daytime sleepiness despite normal sleep onset and total sleep duration. The subjects, as a group, had mild to moderate depressive symptoms, but only two were on antidepressants, which indicates that medication would not be able to explain the abnormalities.

Only one study reported on neurocognitive change: Shi at al. reported that SARS-infected healthcare workers had impaired immediate recall as compared to the control group in the early post-discharge period [36]. After 5 years, however, they improved to the same level as the control group, but showed an increase in wordlist intrusion error. The dropout rate of the follow-up was high (67% retention).

#### Mental health-related healthcare utilisation

A Canadian study reported that, in terms of the number of visits, psychiatric-related healthcare utilisation was the highest among all disciplines in the first year post-discharge [39]. Yip reported that 34.4% of their subjects were still receiving active psychiatric follow-up 10 years post-SARS [46].

### Non-infected healthcare workers

Studies reporting on mental health outcomes of non-infected healthcare workers of SARS-affected regions covered the epidemic period and up until 3 years post-SARS [48–52, 54–62, 64–75]. The most commonly reported dimensions were PTSD, depression, and general psychological distress.

**Fig. 4:**
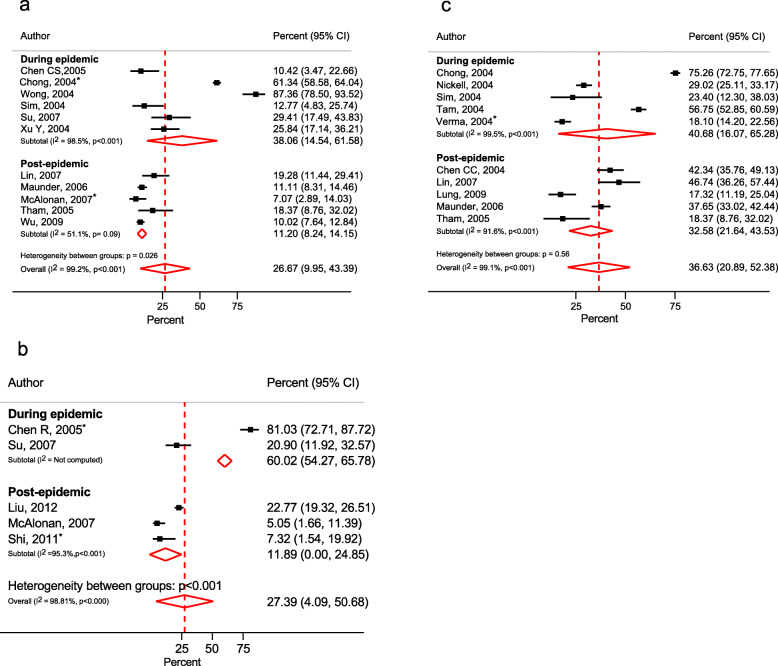
Forest plots of pooled estimates of prevalence of mental health outcomes among healthcare workers of SARS-affected regions. **a** Forest plot of pooled estimate of prevalence of probable PTSD among healthcare workers; **b** Forest plot of pooled estimate of prevalence of probable depression among healthcare workers; **c** Forest plot of pooled estimate of prevalence of significant psychological distress among healthcare workers. Note: ^*^Prevalence calculated from mean and standard deviation using Monte Carlo simulation

The estimated point prevalence of probable PTSD among non-infected healthcare workers from 11 available studies [48, 50, 51, 56, 57, 61, 65, 67, 69, 72, 73] was 26.7% (95% CI: 10.0–43.4%, I^2^ = 99.2%; *n* = 2791). Subgroup analysis showed that the point prevalence during and after the epidemic was 38.1% (95% CI: 14.5–61.6%, I^2^ = 98.5%; from six studies with total *n* = 1579), and 11.2% (95% CI: 8.2%-14,2%, I^2^ = 51.1%; from five studies with total *n* = 1212) respectively. There was a significant difference between the prevalence of the two periods (*p* = 0.035) (Fig. 4a.

The estimated point prevalence of probable depressive disorder among non-infected healthcare workers from five studies [36, 56, 57, 74, 93] was 27.4% (95% CI: 4.1–50.7%, I^2^ = 98.8%; *n* = 872), Subgroup analysis showed that the point prevalence after the epidemic was 11.9% (95% CI: 0–24.9%, I^2^ = 95.3%; from three studies with total *n* = 689). There was an insufficient number of studies to conduct a subgroup analysis of the data from the epidemic period (Fig. 4b).

The estimated point prevalence of significant general psychological distress among non-infected healthcare workers during and after the epidemic was 40.7% (95% CI: 16.1–65.3%, I^2^ = 99.5%; from five studies [49, 50, 62, 63, 65] with total *n* = 2786), and 32.6% (95% CI: 21.6–43.5%, I^2^ = 91.6%; from five studies [67–70, 72] with total *n* = 915) respectively. The prevalence of the two periods were not significantly different (*p* = 0.556) (Fig. 4c.

Only two studies used clinical interviews as part of their diagnostic tools, and both yielded a low rate of psychological distress among their subjects. Su et al. reported that the prevalence of PTSD and depression among their sample was 0 and 12%, respectively, around the end of the SARS outbreak [56]. The study by Lancee et al., which captured a subset of the sample of the study by Maunder et al. (2006), also reported a low rate of PTSD (1.5%) and post-SARS mental illness episodes (6.8%) 1 to 2 years after SARS [71].

### General public

Nineteen studies examined the effect of SARS on the general public [37, 53, 76–91]. The lack of a pre-SARS baseline and long-term longitudinal data limited the usefulness of many of these studies in assessing the mental health impact of SARS among the general public. Furthermore, the sub-populations in these studies, which included hospitalised patients with non-SARS illnesses, college students, pregnant women and people under quarantine, were too heterogenous. We thus considered it inappropriate to pool the estimates from these studies. Two studies with a pre-SARS baseline showed an increase in anxiety and depressive symptoms during the epidemic [76, 80]. A series of Hong Kong population-wide studies by Leung et al. showed that the trend of the population’s anxiety levels followed the trend of the number of daily new cases [77]. One small prospective cohort study on college students showed that the subjects’ anxiety levels increased markedly in the early phase of the epidemic but gradually reduced over time [53].

It was unclear whether there were any increases in clinically significant outcomes among the public. A Hong Kong study that used longitudinal death registry data reported a significantly increased elderly suicide rate in the year of the epidemic, against a downward trend in previous years, with an unusual peak around the peak of the epidemic. The elderly suicide rate did not return to baseline in 2004 [90]. A study from Taiwan, which used a population insurance database with a high population coverage to study the effect of the global financial crisis of 2008–2009 on the incidence of depression, incidentally found a significant upward deviation from the expected incidence of depression starting from July 2003 (right after the epidemic) [91]. The presentation of this study’s methodology and analysis was limited, however, and the results should therefore be treated with caution.

### Sensitivity analysis

Age (≤40 years vs > 40 years) was not found to be a significant factor explaining the heterogenicity of the results. The prevalence of PTSD among SARS survivors post-discharge from studies that used clinical interviews and self-reported screening instruments were not significantly different (see Supplementary Figures 4–8).

### Factors associated with mental health outcome

Due to the diversity of outcome measures and statistical methods, a quantitative analysis could not be conducted. We present the results qualitatively below:

#### Gender

Twenty-four studies had data that allowed an analysis of gender differences in mental health outcomes among different populations. Of these, nine were on SARS patients [27, 28, 30–33, 43, 45, 46]. The results were not consistent: only three out of nine studies found a significant gender effect on mental health outcomes [27, 31, 45]. Of the ten studies that covered non-infected healthcare workers [49–51, 62, 66, 72–75, 93], the results were also inconsistent: six did not reveal a significant gender difference in mental health outcomes [49, 51, 66, 73, 74, 93]. Among the general public, one population-wide telephone survey from Hong Kong [77] reported that women had a higher anxiety level than men during the outbreak, but the anxiety levels of women dropped to that of men 6 months post-SARS. By contrast, a survey on Beijing college students reported a higher level of anxiety among male students during the epidemic [78]. A Canadian study on quarantined individuals during the outbreak and a Taiwanese population-wide telephone survey post-SARS did not find gender differences in mental health outcomes [87, 88].

#### Healthcare workers

Nine studies reported on the association between healthcare worker status and mental health outcomes among SARS patients. One study during the acute phase reported that healthcare workers had a similar stress level as non-healthcare worker patients [22]. Three studies by Cheng et al. found that healthcare worker status was associated with an increase in anxiety, depression and general psychiatric morbidities among patients in the first few months post-discharge, after adjusting for gender and ICU admission status [27, 28, 38]. Wu et al. reported that healthcare worker patients had worse symptoms of hyperarousal and intrusion, two of the PTSD symptom domains, but not avoidance, anxiety or depressive symptoms [29]. Lam et al. reported that 3 to 4 years post-SARS, healthcare worker patients were at significantly higher risk of having a DSM4 Axis I diagnosis [43]. By contrast, while Mak et al.’s two-year follow-up study also found that healthcare workers were at an increased risk of PTSD from univariate analysis, the effect ceased to be significant after adjusting for gender [45]. Yip’s ten-year post-SARS follow-up of Mak et al.’s cohort did not find healthcare worker status to be a significant predictor of PTSD from univariate analysis [46]. Kwek et al.’s Singaporean study found a trend of worse HRQoL, PTSD symptoms, anxiety and depressive symptoms among patients who were healthcare workers, but this did not reach statistical significance [25].

#### Occupational exposure to SARS

Fifteen studies examined occupational exposure to SARS as a predictor of mental health outcomes [48, 50, 52, 54, 56–58, 60, 62, 63, 66, 69, 70, 72–75]. The studies used diverse methods to quantify occupational exposure. Studies that compared levels of exposure at an institutional level (i.e. SARS hospital vs non-SARS hospital) and a work-unit level (i.e. SARS units vs non-SARS units) consistently reported an increase in mental health morbidities among healthcare workers who worked in high-risk environments during and after SARS. Styra et al. reported that working in a high-risk unit was a significant risk factor for high post-traumatic stress symptoms (univariate odd ratio = 3.2) during the epidemic, even after adjustment for the number of SARS patients cared for [66].

Maunder et al. compared the mental health outcomes of healthcare workers in SARS-hit Toronto and SARS-free Hamilton nearby. A significantly higher rate of significant psychological distress and burnout, as well as a marginally higher number of probable PTSD cases (13.8% vs 8.6%; *p* = 0.06), were found among Toronto healthcare workers 1 to 2 years after the epidemic. The Toronto group also exhibited an increase in maladaptive behaviour since the SARS epidemic (21% vs 8.1%; *p* < 0.001%) [72].

A study from Taiwan reported that the proportion of general psychological distress followed risk of occupational exposure at an institutional level (i.e. SARS hospital > 2 general hospitals > psychiatric hospital) during the early post-SARS period [70]. McAlanon et al. showed that high-risk unit staff had worse anxiety and depression 1 year post-SARS [57]. Lin et al. reported that emergency staff had more PTSD symptoms than psychiatric staff post-SARS, but found no difference in general psychological distress [69]. The three studies from the same Beijing sample found depressive symptoms (odd ratio (OR) = 2.22, *p* = 0.05), PTS symptoms (OR = 2.09, *p* < 0.05), and alcohol abuse symptoms to be positively associated with exposure to high-risk locations [73–75].

When exposure was measured in terms of direct contact with SARS patients, it was also reported as a significant risk factor for worse mental health outcomes among healthcare workers during the epidemic in seven out of eight studies [50, 52, 54, 58, 60, 63, 66], with Tam et al.’s study being the only exception [62].

#### SARS impact on close social circles

Eleven studies examined whether infection or death within close social circles of subjects increased adverse mental health outcomes [20, 28, 29, 31, 32, 43, 45, 46, 51, 73–75]. Lee et al. reported that, among patients, having family members infected by SARS was a marginally significant predictive factor (*p* = 0.06) of having psychotic disorder during the treatment phase [20]. Follow-up studies of patients that covered the early post-discharge period generally agreed that infection or death among friends or family significantly increased depression and PTSD symptoms [28, 29, 31, 32]. Studies with a longer post-SARS follow-up duration (Lam et al., Mak et al. and Yip), also showed such a trend of association, but the association did not reach statistical significance [43, 45, 46]. In relation to non-infected healthcare workers, two studies from China reported that the death of relatives or friends from SARS was positively associated with PTSD features 3 years post-SARS, but not depressive or alcohol abuse symptoms [32, 73–75].

#### Physical complications

Some SARS survivors suffered residual impairments in lung function for varying periods of time. Hui et al. reported that impaired lung function was not associated with the mental health domain of HRQoL at 12 months post-discharge [26]. The other key physical complication among SARS survivors was avascular necrosis, which resulted from high-dose steroid use to suppress cytokine storm, a deadly complication of SARS [94]. Four studies reported an association between avascular necrosis and mental health outcome [31, 43, 45, 46]. Three studies (including two studies from Mak et al. and Yip) found that avascular necrosis was a significant predictor of PTSD. Lam et al. did not find such an association, but avascular necrosis was rare among their subjects (only three out of 181, and two of those had a psychiatric diagnosis 3 to 4 years post-SARS) [43].

#### ICU admission

Five studies explored the effect of ICU admission on post-discharge mental health outcomes of patients [25–27, 43, 46]. None of the studies found that ICU admission was a significant factor in post-SARS mental health morbidities.

#### Pre-existing mental health problems

The presence or absence of pre-SARS mental health issues was not commonly reported in the reviewed studies, and some studies excluded subjects with known psychiatric illness. Only two studies examined whether pre-SARS psychiatric illness predicted mental health outcomes among patients: Wu et al. found that pre-SARS psychiatric consultations increased all PTSD symptom domains, as well as depressive and anxiety symptoms [29]. Hong et al. did not find pre-SARS psychiatric illness to be a significant factor for PTSD outcomes, although only one subject in the cohort had a known, pre-existing psychiatric illness, and that subject developed PTSD during follow-up [31].

Two studies examined whether pre-SARS psychiatric illness predicted mental health outcomes among non-infected healthcare workers, and both showed that it was a significant factor: Su et al. reported that, among the high exposure risk nurse group, six out of 20 (30%) of those with a past history of depression developed depressive episodes by the end of SARS, as compared to only 7.3% of those who did not have a history of depression [56]. In the Canadian study by Lancee et al., 18% of those with a pre-existing psychiatric condition developed new episodes of mental disorders within 1 to 2 years post-SARS, as compared to only 2% of those who did not have pre-SARS psychiatric illness [71].

#### Organisational support

Two studies examined the relationship between perceived organisational support and mental health outcomes of healthcare workers: Fiksenbaum et al. reported that a lower level of perceived organisational support was positively associated with burnout [60]. Maunder et al. reported that doubts about protective equipment and dissatisfaction with the system were positively associated with PTSD symptoms [54].

#### Perceived stigmatisation

Six studies examined the relationship between perceived stigmatisation and mental health outcome. Lam et al. reported an odds ratio of 2.92 for perceived stigmatisation and psychiatric diagnosis 3 years post-SARS recovery [43]. Mak et al.’s study, which was also from Hong Kong, reported a significantly higher rating of perceived stigmatisation among SARS survivors with PTSD 2 years after SARS [45]. Four studies reported an association between perceived stigmatisation and mental health outcomes among non-infected healthcare workers. Three studies reported on the association between stigmatisation and PTS symptoms, and all were positive [54, 64, 72]. Koh et al. also reported a trend of positive association between perceived stigmatisation and symptoms of burnout among healthcare workers, but not general psychological distress. Verma et al.’s study, also conducted in Singapore, reported higher perceived stigmatisation among those with higher general psychological distress [63].

#### Effect of quarantine

The evidence for the association between quarantine and mental health outcomes was mixed among healthcare workers. One study reported that quarantine was associated with an increase in burnout during the outbreak [60]. The Beijing studies reported that 3 years post-SARS, the quarantine experience increased the risk of having probable depression (OR 4.9, *p* < 0.001) [74] and PTSD (OR 2.09, *p* = 0.05) [73]. Quarantine was also positively associated with alcohol use [75]. Two other studies, however, did not find such an association. Styra et al. found that quarantine was a significant predictor of post-traumatic stress symptoms in univariate analysis, but this effect disappeared in multivariate analysis [66]. Maunder et al. did not find any association between quarantine and mental health outcomes 1 year post-SARS [72]. One study among the general public found that people who underwent a longer quarantine period (> 10 days) had significantly worse post-traumatic stress symptoms, and a trend for worse depressive symptoms (*p* = 0.07) [86].

### Quality of evidence assessment

#### Risk of bias of individual studies

The rating of the methodological quality of each study is listed in Table 1. Overall, 13 and nine of the studies were rated as being of poor and good quality, respectively, by both reviewers. Common potential sources of bias included biased sampling (e.g. convenient sample with low response rate), small sample size, lack of blinding in the assessment process, and risk factors being measured at the same time as outcome measurements.

#### Publication bias

The visualisation of funnel plots revealed a publication bias for the prevalence of general psychiatric distress among healthcare workers, with studies more inclined to publish a higher prevalence. Publication bias was also noted for studies that published on the prevalence of PTSD among healthcare workers, as well as among patients, with no inclination towards either lower or higher prevalence. Funnel plots of studies for anxiety and depression among patients did not reveal any publication bias. Egger’s test found insufficient evidence for small study effects in studies on the prevalence of general psychological distress among healthcare workers (*p* = 0.069), and PTSD among patients (*p*-value = 0.982). However, this test suggested small study effects in the prevalence of anxiety (*p*-value = 0.009), depression (*p*-value = 0.044), and PTSD among patients (*p*-value = 0.003) (see Supplementary Figures 9–13).

#### Inconsistency across studies

The inconsistency across studies was generally high, as evidenced by the high heterogeneity across most outcomes, except for the pooled estimate of mean scores of the mental health and social functioning domains of SF-36. Sensitivity analysis showed that, among SARS patients, high heterogeneity was found only for studies with mean age ≤ 40 years for anxiety (see Supplementary Figure 4) and depression (see Supplementary Figure 5) but not for studies with mean age > 40 years. However, for PTSD, there was high heterogeneity for both age groups (see Supplementary Figure 6) and for cross-sectional studies, but minimal heterogeneity for prospective studies (see Supplementary Figure 7). In addition to this, among SARS patients, there was high heterogeneity for PTSD studies irrespective of whether the outcome was assessed via clinical interview or self-report/questionnaire (see Supplementary Figure 8).

## Discussion

In preparing for the mental health crisis that is likely to accompany and follow on from the covid-19 pandemic, an in-depth understanding of the mental health effects of SARS will likely provide key insights. We argue that, instead of mixing evidence derived from different events of infectious disease outbreak, focusing on a single event will preserve the historical and socio-cultural context, which will in turn facilitate interpretation and generalisation of the results. The differences between SARS and covid-19, however, are not to be understated. A key difference, for example, lies in the fact that SARS patients generally had more severe symptoms and were universally hospitalised. The 17-year gap between them also resulted in changes in the medical and socio-cultural contexts (see commentary by Sommer and Bakker) [95].

### SARS patients

Our results showed that mental health problems were common among SARS survivors. The prevalence rates of anxiety, depression, and PTSD from our analysis are comparable with an earlier meta-analysis that included studies from SARS, MERS and covid-19 [10]. Subgroup analysis of a recent meta-analysis by Yuan et al. on the prevalence of post-infectious outbreak PTSD (which includes studies from SARS) concluded that the prevalence of PTSD was 18.6% within 6 months post-infection, and 28.8% beyond 6 months of infection [12]. However, the different mix of studies in the subgroups (i.e. the fact that more studies in the within-six-months group came from covid-19, while studies from SARS predominated in the beyond-six-months group) makes interpretation of the difference between the subgroups difficult. Our review additionally introduces the first meta-analytic evidence demonstrating the chronicity of the mental health burden among SARS survivors in terms of HRQoL and psychiatric morbidities, including but not limited to PTSD. Despite the very different context between the two novel coronavirus outbreaks, our findings suggest similarities between post-SARS and post-covid-19 neuropsychiatric sequalae: the prevalence of psychiatric morbidities among SARS patients within the first 6 months post-recovery was remarkably similar to that of covid-19 patients in their early post-recovery period, and survivors of both infections reported prolonged excessive fatigue [96]. In the case of SARS, it is intriguing that a substantial proportion of patients still suffered from excessive fatigue years after recovery from SARS, which cannot be explained by the presence of other psychiatric disorders. We have little idea of the biological aetiology of chronic mental health sequelae and fatigue post-SARS, although what we do know is that the SARS-CoV virus can be isolated from the brains of SARS patients [97], just as it is possible to isolate SARS CoV-2 from the brains of covid-19 patients [98]. How the presence of SARS-CoV viruses in the brain affects patients’ mental health, however, is a question that remains to be answered. Overall, while we cannot conclude based on these resemblances that post-covid-19 neuropsychiatric sequalae will follow the same trajectory as that of SARS, our findings suggest that preparations will be needed to address the long-term mental health needs of covid-19 patients.

### Healthcare workers

There are discrepancies among studies concerning healthcare worker status as a risk factor for poor mental health outcomes of SARS patients, and a definitive conclusion cannot be drawn from our analysis. Psychological distress among healthcare workers was high during the epidemic, but the level of psychiatric morbidities returned to a lower level afterwards. While these results are encouraging, one should not underestimate the impact of mental health consequences for healthcare workers on our healthcare systems: Lam et al. reported that 9/43 of healthcare worker patients with a psychiatric diagnosis left health care-related work (21%), as compared to 4.7% among those without a psychiatric diagnosis [43]. Maunder et al. reported that healthcare workers of SARS-hit Toronto had a significantly higher rate of missing work shifts due to stress or illness compared to SARS-free Hamilton since SARS (21.6% vs 12.6%) [72]. Given the large numbers of healthcare workers who are involved in the care of covid-19 patients and/or suffer from the disease themselves, the impact of the mental health morbidities of this population on healthcare systems will be, like the covid-19 pandemic itself, in uncharted waters. The increase in service demand, met with substantial manpower loss, may tip the balance in regions where healthcare resources are already overstretched. In preparing for the aftermath of covid-19, it is noted that occupational exposure and stigmatisation were consistently reported as risk factors for poor mental health outcomes among healthcare workers, suggesting that these will be issues that require close attention. The recent meta-analysis by Kisley et al. has already demonstrated that direct contact with infected patients during novel viral outbreaks (including SARS) is a significant risk factor for higher acute or post-traumatic stress and psychological distress [11], but this study used a restricted definition of exposure (i.e. direct contact) and did not differentiate between its acute and sustained effect. Our qualitative analysis suggests that the effect of working in high-risk venues might persist beyond the epidemic period.

### General public

While the evidence converges in suggesting that the general public experienced an increase in anxiety levels during the SARS epidemic, no conclusive evidence can be established regarding enduring or clinically significant mental health effects on the public due to the scarcity of longitudinal data spanning from the pre-SARS to post-SARS period. Other factors also affect the interpretation of available statistics: for example, the psychiatric bed occupancy of the greater Toronto area paradoxically dropped sharply during the early phase of SARS, but this may merely be a reflection of the obstruction of access to mental health services during an infectious outbreak [99]. A point of concern that needs to be highlighted, however, is the report from Hong Kong of the increase in suicide rates among older adults during the SARS epidemic [90]. Although this evidence was from a single study and from only one region, it was methodologically robust. A qualitative study has further identified several common factors among SARS-related suicides in older adults in Hong Kong: fear of contracting SARS, social isolation, disruption of normal social life, and the burden of existing long-term illnesses [100]. These factors are likely also present, if not to a greater extent, in the current covid-19 epidemic.

### Stigmatisation

Stigmatisation was consistently reported to be associated with poor mental health outcomes among SARS patients and healthcare workers. Many SARS patients and their family members, healthcare workers, and residents from SARS-hit neighbourhoods encountered discrimination in various aspects of their lives [37, 64, 101]. The stigma against SARS patients, in particular, persisted years after the outbreak. One account reported that SARS survivors in Hong Kong were denied the opportunity to donate blood 10 years post-epidemic [102]. In the covid-19 context, there have been reports of stigmatisation of covid-19 patients and healthcare workers [103], which is unfortunate but unsurprising. De-stigmatisation appears crucial in mitigating post-covid mental health sequelae, but this would require more than evidence-based public education, as stigmatisation can be unintentionally institutionalised. For example, some researchers have argued that the establishment of special post-SARS clinics has in fact perpetuated SARS-related stigmatisation [104]. This highlights the importance of being mindful of this issue in all aspects of post-covid-19 policy planning.

### Strengths and limitations

To the best of our knowledge, this is the first comprehensive systematic review of evidence concerning the effect of SARS on mental health across multiple study populations and timeframes that also captures non-English written studies. This review is mainly limited by the methodologies and quality of the included studies. The studies included were primarily cross-sectional in design, and the association of risk factors and mental health outcomes found in these studies thus cannot be concluded as causal in nature. The few longitudinal studies tended to have small sample sizes. The methodological quality of the studies was generally in the poor to fair range, indicating significant to moderate risk of bias. We did not exclude low quality studies due to the scarcity of studies for specific outcomes, and also because we wanted to represent the available literature comprehensively. The inclusion of low-quality studies in the context of the small number of studies available for data synthesis for each outcome, however, limits the certainty of the results of the review. Another limitation of this study is that, due to the limited numbers of studies included in each meta-analysis and the availability of variables, we could only investigate heterogeneity among studies by mean age, study design, and outcome measurement tool. Since most of the studies used self-rating instruments for measuring outcomes, and the tools used for each outcome were diverse, it is reasonable to suspect that the diverse measurement tools could have introduced heterogeneity to the results of our meta-analysis. We could not investigate whether this was indeed the case, however, due to the limited number of studies available for each outcome measure. Another limitation is that, while we assessed publication bias for outcomes with ≥10 studies, the number of studies included in the funnel plots ranged from 10 to 12, which could limit the power to test for real asymmetry and publication bias. Taking these factors into account, the certainty of the body of evidence of this review is considered to be low.

## Conclusion

Our review suggests that there was a high prevalence of psychiatric morbidities and HRQoL impairment beyond the early post-recovery period in 2002–2003 SARS outbreak survivors. This should be considered a predictive indicator for what may be expected among covid-19 patients, and preparation for this thus needs to be considered. Although our results suggest that healthcare workers are resilient against clinically significant mental health effects after an epidemic, efforts to support healthcare workers, especially those working in high-risk venues, are essential to prevent widespread workforce loss. A significant knowledge gap remains regarding the biological link between SARS-CoV viruses and long-term neuropsychiatric morbidities of patients, warranting robust methodological investigation in relation to SARS-Cov-2. Stigmatisation against patients and healthcare workers may result in a secondary impact on mental health, and should be carefully addressed in the covid-19 era. Overall, due to the limitations from the methodological constraints of the included studies, as well as the relatively small number of studies for each outcome measure and the high degree of heterogeneity in most outcome measures, the certainty of the body of evidence is low.

## Supplementary Information

**Additional file 1.** Full search terms and search history.

**Additional file 2.** List of excluded studies with reasons.

**Additional file 3.** Supplementary figures.

**Additional file 4.** PRISMA checklist.

## Data Availability

The datasets used and/or analysed in the current study are available from the corresponding author on reasonable request.

## Abbreviations

CI: Confidence interval
IQR: Interquartile range
HRQoL: Health-related quality of life
OR: Odd ratio
PTSD: Post-traumatic stress disorder
SARS: Severe Acute Respiratory Syndrome
SD: Standard deviation
SF-36: 36-items Short Form Survey
