# Supplementary material for: History for some or lesson for all? A systematic review and meta-analysis on the immediate and long-term mental health impact of the 2002–2003 Severe Acute Respiratory Syndrome (SARS) outbreak
Source: BMC Public Health. 2021 Apr 7;21:670. doi: 10.1186/s12889-021-10701-3 (PMC8025448; doi:10.1186/s12889-021-10701-3)
Supplement: Supplementary file 1 — Additional file 1. Full search terms and search history. [file 12889_2021_10701_MOESM1_ESM.pdf]

Additional file 1: Full search terms and search history

Index

Page no.	Database
p2 - p3	Pubmed
p4- p5	Medline
p6	Embase
p7	PsycInfo
p8	Web of Science Core Collection
p9	HKU Scholar Hub
p10	ProQuest CUHK
p11	The Hong Kong College of Psychiatrists Dissertation database
p12	CNKI (Master)
p13	CNKI (Doctoral)
p14	Digital Dissertation Concortium
p15	NDLTDTaiwan
p16	NCLTaiwan
p17	Airiti Library
p18	NUS datebase
p19	Thesis Canada

Search ID	Search term 1	AND/OR	Search term 2	Filters	Search date	Number of result
pubmed_01	**((severe acute respiratory syndrome[MeSH Terms]) OR (SARS[Title/Abstract]) OR (Respiratory Syndrome, Acute, Severe[Title/Abstract]) OR (Respiratory Syndrome, Severe Acute[Title/Abstract]))**	AND	**((Psychological Distress[MeSH Terms]) OR (Psychological stress[Title/Abstract]) OR (Psychological effect [Title/Abstract]) OR (Emotional effect [Title/Abstract]) OR (Emotional distress[Title/Abstract]) OR (Emotional stress[Title/Abstract]) OR (Psychological impact[Title/Abstract]) OR (Emotional**	**Human, English, Chinese**	4/5/2020	34
pubmed_02	**((severe acute respiratory syndrome[MeSH Terms]) OR (SARS[Title/Abstract]) OR (Respiratory Syndrome, Acute, Severe[Title/Abstract]) OR (Respiratory Syndrome, Severe Acute[Title/Abstract]) NOT (covid[Title/Abstract]) NOT(SARS-COV-2 [Title/Abstract]))**	AND	((Mental Disorders [MeSH]) OR (Psychophysiologic Disorders [MeSH]) OR (Mental Disorders[Title/Abstract]) OR (Psychiatric Disorders [Title/Abstract]) OR (Psychiatric Diagnosis [Title/Abstract]) OR (Psychiatric Diagnoses [Title/Abstract]) OR (Psychophysiological Disorder* [Title/Abstract]) OR (Psychosomatic	**Human, English, Chinese**	7/5/2020	120
pubmed_03	**((severe acute respiratory syndrome[MeSH Terms]) OR (SARS[Title/Abstract]) OR (Respiratory Syndrome, Acute, Severe[Title/Abstract]) OR (Respiratory Syndrome, Severe Acute[Title/Abstract]))**	AND	((Mental Health [MeSH]) OR (Quality of Life [MeSH]) or (social stigma[MeSH]) OR (Mental Health [Title/Abstract]) OR (Mental Well-being[Title/Abstract]) OR (Mental Wellness[Title/Abstract]) OR (Quality of Life [Title/Abstract]) OR (stigm* [Title/Abstract]))	**Human, English, Chinese**	4/5/2020	84
pubmed_04	**((severe acute respiratory syndrome[MeSH Terms]) OR (SARS[Title/Abstract]) OR (Respiratory Syndrome, Acute, Severe[Title/Abstract]) OR (Respiratory Syndrome, Severe Acute[Title/Abstract]))**	AND	((Depression[MeSH]) OR (Depression [Title/Abstract]) OR (Depressive Symptoms[Title/Abstract]) OR (Depressive Disorder[MeSH]) OR (Depressive Disorder[Title/Abstract]) OR (Depress* [Title/Abstract]) OR (Mood Disorders[MeSH]) OR (Mood Disorders[Title/Abstract]) OR (Affective Disorders[Title/Abstract]) OR (Bipolar [Title/Abstract]))	**Human, English, Chinese**	5/5/2020	57
pubmed_05	**((severe acute respiratory syndrome[MeSH Terms]) OR (SARS[Title/Abstract]) OR (Respiratory Syndrome, Acute, Severe[Title/Abstract]) OR (Respiratory Syndrome, Severe Acute[Title/Abstract]) NOT (covid[Title/Abstract]) NOT(SARS-COV-2 [Title/Abstract]))**	AND	(Anxiety Disorders [MeSH]) OR (Anxiety [MeSH]) OR (Anxiety Disorders [Title/Abstract]) OR (Anxiety [Title/Abstract]) OR (phobi* [Title/Abstract]) OR (agoraphobi* [Title/Abstract]) OR (panic [Title/Abstract])	**Human, English, Chinese**	7/5/2020	91
pubmed_06	**((severe acute respiratory syndrome[MeSH Terms]) OR (SARS[Title/Abstract]) OR (Respiratory Syndrome, Acute, Severe[Title/Abstract]) OR (Respiratory Syndrome, Severe Acute[Title/Abstract]))**	AND	(Stress Disorders, Post-Traumatic [MeSH]) OR (Stress Disorders, Post-Traumatic [Title/Abstract]) OR (Stress Disorder, Post Traumatic [Title/Abstract]) OR (Post-Traumatic Stress Disorder [Title/Abstract]) OR (Posttraumatic Stress Disorder [Title/Abstract]) OR (PTSD [Title/Abstract])	**Human, English, Chinese**	4/5/2020	30
pubmed_07	**((severe acute respiratory syndrome[MeSH Terms]) OR (SARS[Title/Abstract]) OR (Respiratory Syndrome, Acute, Severe[Title/Abstract]) OR (Respiratory Syndrome, Severe Acute[Title/Abstract]))**	AND	(Sleep Initiation and Maintenance Disorders [MeSH]) OR (Sleep [MeSH]) OR (Insomnia [Title/Abstract]) OR (Sleep Disorders [MeSH]) OR (Sleep Disorders [Title/Abstract]) OR (Sleep Disturbance [Title/Abstract])	**Human, English, Chinese**	4/5/2020	13
pubmed_08	**((severe acute respiratory syndrome[MeSH Terms]) OR (SARS[Title/Abstract]) OR (Respiratory Syndrome, Acute, Severe[Title/Abstract]) OR (Respiratory Syndrome, Severe Acute[Title/Abstract]))**	AND	(Substance-Related Disorders [MeSH]) (Alcoholism [MeSH]) OR (Substance Abuse [Title/abstract]) OR (Substance Use Disorders [Title/abstract]) OR (Drug Abuse [Title/abstract]) OR (Alcoholism [Title/abstract]) OR (Alcohol abuse [Title/abstract]) OR (Alcohol [Title/abstract])	**Human, English, Chinese**	6/5/2020	20

pubmed_09	**((severe acute respiratory syndrome[MeSH Terms]) OR (SARS[Title/Abstract]) OR (Respiratory Syndrome, Acute, Severe[Title/Abstract]) OR (Respiratory Syndrome, Severe Acute[Title/Abstract]))**	AND	(Suicide [MeSH]) OR (Suicide, Attempted [MeSH]) OR (Suicide, Completed [MeSH]) OR (Suicide [Title/Abstract]) OR (Suicide Attempt [Title/Abstract]) OR (Suicidal Idea [Title/Abstract]) OR (Suicidality [Title/Abstract]) OR (Suicidal Ideation [Title/Abstract]) OR (Self-harm [Title/Abstract])	**Human, English, Chinese**	5/5/2020	13
pubmed_10	**((severe acute respiratory syndrome[MeSH Terms]) OR (SARS[Title/Abstract]) OR (Respiratory Syndrome, Acute, Severe[Title/Abstract]) OR (Respiratory Syndrome, Severe Acute[Title/Abstract]) NOT (covid[Title/Abstract]) NOT(SARS-COV-2 [Title/Abstract]))**	AND	(Health Personnel [MeSH]) OR (Allied Health Personnel [MeSH]) OR (Health Personnel [Title/Abstract]) OR (Allied Health Personnel [Title/Abstract]) OR (Health Workers [Title/Abstract]) OR (Healthcare Workers [Title/Abstract]) OR (Health Care Workers [Title/Abstract]) OR (Healthcare Assistants [Title/Abstract]) OR (Health Care Assistants [Title/Abstract]) OR (Hospital Workers [Title/Abstract]) OR (Hospital Employees [Title/Abstract]) OR (Hospital Staff [Title/Abstract]) OR (Healthcare Professionals [Title/Abstract]) OR (Health Care Professionals [Title/Abstract]) OR (Healthcare Providers [Title/Abstract]) OR (Health Care Providers [Title/Abstract]) OR (Frontline Workers [Title/Abstract])	**Human, English, Chinese**	7/5/2020	713
pubmed_11	**((severe acute respiratory syndrome[MeSH Terms]) OR (SARS[Title/Abstract]) OR (Respiratory Syndrome, Acute, Severe[Title/Abstract]) OR (Respiratory Syndrome, Severe Acute[Title/Abstract]))**		(Mentally-ill[Title/Abstract]) OR (Mental Patients[Title/Abstract]) OR (Psychiatric Patients[Title/Abstract])	**Human, English, Chinese**	5/5/2020	0
pubmed_12	**((severe acute respiratory syndrome[MeSH Terms]) OR (SARS[Title/Abstract]) OR (Respiratory Syndrome, Acute, Severe[Title/Abstract]) OR (Respiratory Syndrome, Severe Acute[Title/Abstract]) NOT (covid[Title/Abstract]) NOT(SARS-COV-2 [Title/Abstract]))**	AND	((Psychosis [MeSH]) OR (psychosis [Title/Abstract]) OR (psychotic [Title/Abstract]) OR (schizo* [Title/Abstract]) OR (delusion* [Title/Abstract]))	**Human, English, Chinese**	7/5/2020	12
					Total	1187

Search ID	Search term 1	AND/OR	Search term 2	Filters	Search date	Number of result
ovid_01	exp *Severe Acute Respiratory Syndrome/ or ((SARS or Severe Acute Respiratory Syndrome) not covid* not SARS-Cov-2).ab,kw,ti.	AND	exp *Stress, Psychological/ or exp *Psychological Distress/ OR (Psychological distress or Psychological stress or Emotional distress or Emotional stress or Psychological impact or Emotional impact or Psychological consequence).ab,kw,ti.	Humans and (Chinese or English)	4/5/2020	57
ovid_02	exp *Severe Acute Respiratory Syndrome/ or ((SARS or Severe Acute Respiratory Syndrome) not covid* not SARS-Cov-2).ab,kw,ti.	AND	exp *Mental Disorders/ OR exp *Psychophysiologic Disorders/ OR (Mental Disorders or Psychiatric Disorders or Psychiatric Diagnosis or Psychophysiological Disorders or Psychosomatic Disorders or Mental Morbidities or Mental illness or Neurocognit* or neuropsychi* or cogniti*).ab,kw,ti.	Humans and (Chinese or English)	7/5/2020	95
ovid_03	exp *Severe Acute Respiratory Syndrome/ or ((SARS or Severe Acute Respiratory Syndrome) not covid* not SARS-Cov-2).ab,kw,ti.	AND	exp *Mental Health/ or exp *Quality of Life/ or exp *Social Stigma/ OR (Mental Health OR Quality of Life OR stigm* OR Mental Well-being OR Mental Wellness OR mental health consequences).ab,kw,ti	Humans and (Chinese or English)	7/5/2020	81
ovid_04	exp *Severe Acute Respiratory Syndrome/ or ((SARS or Severe Acute Respiratory Syndrome) not covid* not SARS-Cov-2).ab,kw,ti.	AND	(Mentally-ill OR Mental Patients OR Psychiatric Patients).ab,kw,ti.	Humans and (Chinese or English)	7/5/2020	0
ovid_05	exp *Severe Acute Respiratory Syndrome/ or ((SARS or Severe Acute Respiratory Syndrome) not covid* not SARS-Cov-2).ab,kw,ti.		exp *Depression/ or exp *Depressive Disorder/ or exp *Mood Disorders/ or (Depression or Depressive symptoms or Depressive Disorder or Depress* or Mood Disorder* or Affective Disorder* or Bipolar).ab,kw,ti.	Humans and (Chinese or English)	7/5/2020	61
ovid_06	exp *Severe Acute Respiratory Syndrome/ or ((SARS or Severe Acute Respiratory Syndrome) not covid* not SARS-Cov-2).ab,kw,ti.	AND	exp *Anxiety Disorders / or exp *Anxiety / or (Anixety or Anxiety Disorders Or Phobia OR agoraphobi* OR Panic).ab,kw,ti.	Humans and (Chinese or English)	7/5/2020	37

ovid_07	exp *Severe Acute Respiratory Syndrome/ or ((SARS or Severe Acute Respiratory Syndrome) not covid* not SARS-Cov-2).ab,kw,ti.	AND	exp *Stress Disorders, Post-Traumatic / or (Stress Disorders, Post-Traumatic OR Post-Traumatic Stress Disorder OR Posttraumatic Stress Disorder OR PTSD).ab,kw,ti.	Humans and (Chinese or English)	7/5/2020	25
ovid_08	exp *Severe Acute Respiratory Syndrome/ or ((SARS or Severe Acute Respiratory Syndrome) not covid* not SARS-Cov-2).ab,kw,ti.	AND	exp *"Sleep Initiation and Maintenance Disorders" / or exp *Sleep / or exp *Sleep Disorders / or (Insomnia or Sleep Disorder* or Sleep Disturbance).ab,kw,ti.	Humans and (Chinese or English)	7/5/2020	7
ovid_09	exp *Severe Acute Respiratory Syndrome/ or ((SARS or Severe Acute Respiratory Syndrome) not covid* not SARS-Cov-2).ab,kw,ti.	AND	exp *Substance-Related Disorders / or exp *Alcoholism / or (Substance Abuse or Drug Abuse or Alcoholism or Alcohol abuse or Alcohol*).ab,kw,ti.	Humans and (Chinese or English)	7/5/2020	22
ovid_10	exp *Severe Acute Respiratory Syndrome/ or ((SARS or Severe Acute Respiratory Syndrome) not covid* not SARS-Cov-2).ab,kw,ti.	AND	exp *Suicide / or exp *Suicide, Attempted / or exp *Suicide, Completed / or (Suicide or Suicidal Attempt or Suicidal Idea or Suicidality or Suicidal Ideation or Self-harm or Self harm).ab,kw,ti.	Humans and (Chinese or English)	7/5/2020	11
ovid_11	exp *Severe Acute Respiratory Syndrome/ or ((SARS or Severe Acute Respiratory Syndrome) not covid* not SARS-Cov-2).ab,kw,ti.		exp *Health Personnel / or exp *Allied Health Personnel / or (Health Workers OR Healthcare Workers OR Health Care Workers OR Healthcare Assistants OR Health Care Assistants OR Hospital Workers OR Hospital Employees OR Hospital Staff OR Healthcare Professionals OR Health Care Professionals Healthcare Providers OR Health Care Providers OR Frontline Workers OR Doctors OR Physicians OR Nurses).ab,kw,ti.	Humans and (Chinese or English)	7/5/2020	640
ovid_12	exp *Severe Acute Respiratory Syndrome/ or ((SARS or Severe Acute Respiratory Syndrome) not covid* not SARS-Cov-2).ab,kw,ti.	AND	exp *psychosis/ or (psychosis OR Psychotic or schizo* or delusion*).ab,kw,ti	Humans and (Chinese or English)	7/5/2020	13
					Total	1049

Search ID	Search term 1	AND/OR	Search term 2	Filters	Search date	Number of result
Embase_01	exp *Severe Acute Respiratory Syndrome/ or ((SARS or Severe Acute Respiratory Syndrome) not covid* not SARS-Cov-2).ab,kw,ti.	AND	exp *Stress, Psychological/ or exp *Psychological Distress/ OR (Psychological distress or Psychological stress or Emotional distress or Emotional stress or Psychological impact or Emotional impact or Psychological consequence*).ab,kw,ti.	Humans and (Chinese or English)	7/5/2020	65
Embase_02	exp *Severe Acute Respiratory Syndrome/ or ((SARS or Severe Acute Respiratory Syndrome) not covid* not SARS-Cov-2).ab,kw,ti.	AND	exp *Mental Disorders/ OR exp *Psychophysiologic Disorders/ OR exp *cognitive deficit/ or (Mental Disorders or Psychiatric Disorders or Psychiatric Diagnosis or Psychophysiological Disorders or Psychosomatic Disorders or Mental Morbidities or Mental illness or Neurocognit* or neuropsychi*).ab,kw,ti.	Humans and (Chinese or English)	7/5/2020	134
Embase_03	exp *Severe Acute Respiratory Syndrome/ or ((SARS or Severe Acute Respiratory Syndrome) not covid* not SARS-Cov-	AND	exp *Mental Health/ or exp *Quality of Life/ or exp *Social Stigma/ OR (Mental Health OR Quality of Life OR stigm* OR Mental Well-being OR Mental Wellness OR mental health consequences).ab,kw,ti	Humans and (Chinese or English)	7/5/2020	148
Embase_04	exp *Severe Acute Respiratory Syndrome/ or ((SARS or Severe Acute Respiratory Syndrome) not covid* not SARS-Cov-	AND	(Mentally-ill OR Mental Patients OR Psychiatric Patients).ab,kw,ti.	Humans and (Chinese or English)	7/5/2020	0
Embase_05	exp *Severe Acute Respiratory Syndrome/ or ((SARS or Severe Acute Respiratory Syndrome) not covid* not SARS-Cov-2).ab,kw,ti.	AND	exp *Depression/ or exp *Depressive Disorder/ or exp *Mood Disorders/ or (Depression or Depressive symptoms or Depressive Disorder or Depress* or Mood Disorder* or Affective Disorder* or Bipolar).ab,kw,ti.	Humans and (Chinese or English)	7/5/2020	91
Embase_06	exp *Severe Acute Respiratory Syndrome/ or ((SARS or Severe Acute Respiratory Syndrome) not covid* not SARS-Cov-	AND	exp *Anxiety Disorders / or exp *Anxiety / or (Anixety or Anxiety Disorders Or Phobia OR agoraphobi* OR Panic).ab,kw,ti.	Humans and (Chinese or English)	7/5/2020	79
Embase_07	exp *Severe Acute Respiratory Syndrome/ or ((SARS or Severe Acute Respiratory Syndrome) not covid* not SARS-Cov-	AND	exp *Stress Disorders, Post-Traumatic / or (Stress Disorders, Post-Traumatic OR Post-Traumatic Stress Disorder OR Posttraumatic Stress Disorder OR PTSD).ab,kw,ti.	Humans and (Chinese or English)	7/5/2020	28
Embase_08	exp *Severe Acute Respiratory Syndrome/ or ((SARS or Severe Acute Respiratory Syndrome) not covid* not SARS-Cov-	AND	exp *"Sleep Initiation and Maintenance Disorders" / or exp *Sleep / or exp *Sleep Disorders / or (Insomnia or Sleep Disorder* or Sleep Disturbance).ab,kw,ti.	Humans and (Chinese or English)	7/5/2020	8
Embase_09	exp *Severe Acute Respiratory Syndrome/ or ((SARS or Severe Acute Respiratory Syndrome) not covid* not SARS-Cov-	AND	exp *Substance-Related Disorders / or exp *Alcoholism / or (Substance Abuse or Drug Abuse or Alcoholism or Alcohol abuse or Alcohol*).ab,kw,ti.	Humans and (Chinese or English)	7/5/2020	25
Embase_10	exp *Severe Acute Respiratory Syndrome/ or ((SARS or Severe Acute Respiratory Syndrome) not covid* not SARS-Cov-	AND	exp *Suicide / or exp *Suicide, Attempted / or exp *Suicide, Completed / or (Suicide or Suicidal Attempt* or Suicidal Idea* or Suicidal* or Self-harm or Self harm).ab,kw,ti.	Humans and (Chinese or English)	7/5/2020	11
Embase_11	exp *Severe Acute Respiratory Syndrome/ or ((SARS or Severe Acute Respiratory Syndrome) not covid* not SARS-Cov-2).ab,kw,ti.	AND	exp *Health Personnel / or exp *Allied Health Personnel / or (Health Workers OR Healthcare Workers OR Health Care Workers OR Healthcare Assistants OR Health Care Assistants OR Hospital Workers OR Hospital Employees OR Hospital Staff OR Healthcare Professionals OR Health Care Professionals Healthcare Providers OR Health Care Providers OR Frontline Workers OR Doctors OR Physicians OR Nurses).ab,kw,ti.	Humans and (Chinese or English)	7/5/2020	755
Embase_12	exp *Severe Acute Respiratory Syndrome/ or ((SARS or Severe Acute Respiratory Syndrome) not covid* not SARS-Cov-	AND	exp *psychosis/ or (psychosis OR Psychotic or schizo* or delusion*).ab,kw,ti	Humans and (Chinese or English)	7/5/2020	29
					Total	1373

Search ID	Search term 1	AND/OR	Search term 2	Filters	Search date	Number of result
psycinfo_01	((SARS or "Severe Acute Respiratory Syndrome") not covid not "2019 novel coronavirus" not "SARS-CoV-2" not "2019-nCoV").ab,ti,hw,id,mh.			Humans and (Chinese or English)	6/5/2020	457
					Total:	457

Search ID	Search term 1	AND/OR	Search term 2	Filters	Search date	Number of result
WOS_01	TS=(SARS or "severe acute respiratory syndrome") NOT TS=covid NOT TS="2019 novel coronavirus"	AND	TS=("Psychological distress" OR "emotional distress" OR "Psychological stress " OR "emotional distress"	English, Chinese	6/5/2020	92
WOS_02	TS=(SARS or "severe acute respiratory syndrome") NOT TS=covid NOT TS="2019 novel coronavirus"	AND	TS=("Mental Disorder*" or "Psychiatric Disorder*" or "Psychiatric Diagnosis" or "Psychiatric Diagnoses" or	English, Chinese	6/5/2020	181
WOS_03	TS=(SARS or "severe acute respiratory syndrome") NOT TS=covid NOT TS="2019 novel coronavirus"	AND	TS=("Mental Health" OR "Mental Well-being" OR "Mental Wellness" OR "Quality of life" OR "Mental health	English, Chinese	6/5/2020	141
WOS_04	TS=(SARS or "severe acute respiratory syndrome") NOT TS=covid NOT TS="2019 novel coronavirus"	AND	TS=(Mentally-ill OR Mental Patients OR Psychiatric Patients)	English, Chinese	6/5/2020	99
WOS_05	TS=(SARS or "severe acute respiratory syndrome") NOT TS=covid NOT TS="2019 novel coronavirus"	AND	TS=(Psychosis or Psychotic or schizo* or delusion*)	English, Chinese	6/5/2020	64
WOS_06	TS=(SARS or "severe acute respiratory syndrome") NOT TS=covid NOT TS="2019 novel coronavirus"	AND	TS=(Neuropsych* OR neurocog* OR cognitive)	English, Chinese	6/5/2020	64
WOS_07	TS=(SARS or "severe acute respiratory syndrome") NOT TS=covid NOT TS="2019 novel coronavirus"	AND	TS=(Depress* or "Mood Disorder*" or "Affective Disorder*" or Bipolar)	English, Chinese	6/5/2020	108
WOS_08	TS=(SARS or "severe acute respiratory syndrome") NOT TS=covid NOT TS="2019 novel coronavirus"	AND	TS=(Anixety Or Phobia OR *phobia OR Panic)	Humans and (Chinese or English)	6/5/2020	35
WOS_09	TS=(SARS or "severe acute respiratory syndrome") NOT TS=covid NOT TS="2019 novel coronavirus"	AND	TS=("Stress Disorders" or "Stress-related disorders" OR "acute stress reaction" or "acute stress disorder" OR "Post-	Humans and (Chinese or English)	6/5/2020	44
WOS_10	TS=(SARS or "severe acute respiratory syndrome") NOT TS=covid NOT TS="2019 novel coronavirus"	AND	TS=(Insomnia or "Sleep Disorder*" or "Sleep Disturbance" or "Sleep quality")	Humans and (Chinese or English)	6/5/2020	7
WOS_11	TS=(SARS or "severe acute respiratory syndrome") NOT TS=covid NOT TS="2019 novel coronavirus"	AND	TS=("Substance-Related Disorder* " or "Substance Abuse" or "Substance use disorder*" or "Drug Abuse" or Alcoholi* or	Humans and (Chinese or English)	6/5/2020	5
WOS_12	TS=(SARS or "severe acute respiratory syndrome") NOT TS=covid NOT TS="2019 novel coronavirus"	AND	TS= (Suicide or 'Suicidal Attempt*' or 'Suicidal Idea*' or Suicidal* or 'Suicidal or Self-harm or 'Self harm')	Humans and (Chinese or English)	6/5/2020	22
WOS_13	TS=(SARS or "severe acute respiratory syndrome") NOT TS=covid NOT TS="2019 novel coronavirus" NOT TS="SARS-CoV-2" NOT TS="2019-nCoV"	AND	TS=("Health Workers" OR "Healthcare Workers" OR "Health Care Workers" OR "Healthcare Assistants" OR "Health Care Assistants" OR "Hospital Workers" OR "Hospital Employees" OR "Hospital Staff" OR "Healthcare Professionals" OR "Health	Humans and (Chinese or English)	6/5/2020	867
					Total:	1729

Search ID	Search term 1	AND/OR	Search term 2	Search date	Number of result
hku_01	**((title:(SARS)) OR (title:(Respiratory syndrome, severe, acute)) OR (abstract:(SARS)) OR (abstract:(Respiratory syndrome, severe, acute)) OR (title:(severe acute respiratory syndrome)) OR (abstract:(severe acute respiratory syndrome))**	AND	(title:(mental OR psychological OR emotional)) OR (abstract:(mental OR psychological OR emotional)) OR (title:(psychiatric OR mental health OR quality of life)) OR (abstract:(psychiatric OR mental health OR quality of life))	10/6/2020	16
hku_02	**((title:(SARS)) OR (title:(Respiratory syndrome, severe, acute)) OR (abstract:(SARS)) OR (abstract:(Respiratory syndrome, severe, acute)) OR (title:(severe acute respiratory syndrome)) OR (abstract:(severe acute respiratory syndrome))**	AND	(title:(mental well-being OR stigma)) OR (abstract:(mental well-being OR stigma))	10/6/2020	0
hku_03	**((title:(SARS)) OR (title:(Respiratory syndrome, severe, acute)) OR (abstract:(SARS)) OR (abstract:(Respiratory syndrome, severe, acute)) OR (title:(severe acute respiratory syndrome)) OR (abstract:(severe acute respiratory syndrome))**	AND	(title:(depression OR mood disorder OR bipolar)) OR (abstract:(depression OR mood disorder OR bipolar)))	10/6/2020	16
hku_04	**((title:(SARS)) OR (title:(Respiratory syndrome, severe, acute)) OR (abstract:(SARS)) OR (abstract:(Respiratory syndrome, severe, acute)) OR (title:(severe acute respiratory syndrome)) OR (abstract:(severe acute respiratory syndrome))**	AND	(title:(Anxiety OR Stress OR PTSD OR Post traumatic)) OR (abstract:(Anxiety OR Stress OR PTSD OR Post traumatic)))	10/6/2020	20
hku_05	**((title:(SARS)) OR (title:(Respiratory syndrome, severe, acute)) OR (abstract:(SARS)) OR (abstract:(Respiratory syndrome, severe, acute)) OR (title:(severe acute respiratory syndrome)) OR (abstract:(severe acute respiratory syndrome))**	AND	(title:(sleep OR insomnia)) OR (abstract:(sleep OR insomnia)))	10/6/2020	31
hku_06	**((title:(SARS)) OR (title:(Respiratory syndrome, severe, acute)) OR (abstract:(SARS)) OR (abstract:(Respiratory syndrome, severe, acute)) OR (title:(severe acute respiratory syndrome)) OR (abstract:(severe acute respiratory syndrome))**	AND	(title:(healthcare workers OR doctors OR nurses)) OR (abstract:(healthcare workers OR doctors OR nurses)))	10/6/2020	31
				Total	114

Search ID	Search term 1	AND/OR	Search term 2	Search date	Number of result
cu_01	**ti(SARS) OR ti(Severe Acute Respiratory Syndrome) OR ti(Severe, Acute, Respiratory, Syndrome) OR ab(SARS) OR ab(Severe Acute Respiratory Syndrome) OR ab(Severe, Acute, Respiratory, Syndrome)**			10/6/2020	16
				Total	16

Search ID	Search term 1	AND/OR	Search term 2	Search date	Number of result
hkcpsych_01	**SARS**			10/6/2020	1
hkcpsych_02	**Severe Acute Respiratory Syndrome**			10/6/2020	1
				Total	2

Search ID	Search term 1	AND/OR	Search term 2	Search date	Number of
cmt_01	**(TI=('SARS'+'Severe Acute Respiratory Syndrome'+'非典型肺炎'+'严重急性呼吸综合症') OR AB=('SARS'+'Severe Acute Respiratory Syndrome'+'非典型肺炎'+'严重急性呼吸综合症'))**	AND	(TI=('Mental'+'Psychological'+'Emotional'+'心理'+'情绪') OR AB=('Mental'+'Psychological'+'Emotional'+'心理'+'情绪'))	10/6/2020	48
cmt_02	**(TI=('SARS'+'Severe Acute Respiratory Syndrome'+'非典型肺炎'+'严重急性呼吸综合症') OR**	AND	(TI=('焦虑'+'忧郁'+'创伤'+'Anxiety'+'Depression'+'Posttraumatic') OR	10/6/2020	33
cmt_03	**(TI=('SARS'+'Severe Acute Respiratory Syndrome'+'非典型肺炎'+'严重急性呼吸综合症') OR**	AND	(TI=('压力'+'睡眠'+'自杀'+'Stress'+'Sleep'+'Suicide') OR	10/6/2020	127
cmt_04	**(TI=('SARS'+'Severe Acute Respiratory Syndrome'+'非典型肺炎'+'严重急性呼吸综合症') OR AB=('SARS'+'Severe Acute Respiratory Syndrome'+'非典型肺炎'+'严重急性呼吸综合症'))**	AND	(TI=('医护'+'护士'+'医生'+'Healthcare'+'Nurse'+'Doctors') OR AB=('医护'+'护士'+'医生'+'Healthcare'+'Nurse'+'Doctors'))	10/6/2020	62
				Total	270

Search ID	Search term 1	AND/OR	Search term 2	Search date	Number of result
cdd_01	**(TI=('SARS'+'Severe Acute Respiratory Syndrome'+'非典型肺炎'+'严重急性呼吸综合症') OR AB=('SARS'+'Severe Acute Respiratory Syndrome'+'非典型肺炎'+'严重急性呼吸综合症'))**	AND	(TI=('Mental'+'Psychological'+'Emotional'+'心理'+'情绪') OR AB=('Mental'+'Psychological'+'Emotional'+'心理'+'情绪'))	10/6/2020	28
cdd_02	**(TI=('SARS'+'Severe Acute Respiratory Syndrome'+'非典型肺炎'+'严重急性呼吸综合症') OR AB=('SARS'+'Severe Acute**	AND	(TI=('焦虑'+'忧郁'+'创伤'+'Anxiety'+'Depression'+'Posttraumatic') OR	10/6/2020	19
cdd_03	**(TI=('SARS'+'Severe Acute Respiratory Syndrome'+'非典型肺炎'+'严重急性呼吸综合症') OR AB=('SARS'+'Severe Acute**	AND	(TI=('压力'+'睡眠'+'自杀'+'Stress'+'Sleep'+'Suicide') OR	10/6/2020	55
cdd_04	**(TI=('SARS'+'Severe Acute Respiratory Syndrome'+'非典型肺炎'+'严重急性呼吸综合症') OR AB=('SARS'+'Severe Acute Respiratory Syndrome'+'非典型肺炎'+'严重急性呼吸综合症'))**	AND	(TI=('医护'+'护士'+'医生'+'Healthcare'+'Nurse'+'Doctors') OR AB=('医护'+'护士'+'医生'+'Healthcare'+'Nurse'+'Doctors'))	10/6/2020	31
				Total	133

Search ID	Search term 1	AND/OR	Search term 2	Search date	Number of result
ddc_01	**((title:(SARS)) OR (title:(Respiratory syndrome, severe, acute)) OR (abstract:(SARS)) OR (abstract:(Respiratory syndrome, severe, acute)) OR (title:(severe acute respiratory syndrome)) OR (abstract:(severe acute respiratory syndrome))**	AND	(title:(mental OR psychological OR emotional)) OR (abstract:(mental OR psychological OR emotional)) OR (title:(psychiatric OR mental health OR quality of life)) OR (abstract:(psychiatric OR mental health OR quality of life))	10/6/2020	0
ddc_02	**((title:(SARS)) OR (title:(Respiratory syndrome, severe, acute)) OR (abstract:(SARS)) OR (abstract:(Respiratory syndrome, severe, acute)) OR (title:(severe acute respiratory syndrome)) OR (abstract:(severe acute respiratory syndrome))**	AND	(title:(mental well-being OR stigma)) OR (abstract:(mental well-being OR stigma))	10/6/2020	0
ddc_03	**((title:(SARS)) OR (title:(Respiratory syndrome, severe, acute)) OR (abstract:(SARS)) OR (abstract:(Respiratory syndrome, severe, acute)) OR (title:(severe acute respiratory syndrome)) OR (abstract:(severe acute respiratory syndrome))**	AND	(title:(psychosis OR depression OR anxiety) OR (abstract:(psychosis OR depression OR anxiety))	10/6/2020	0
ddc_04	**((title:(SARS)) OR (title:(Respiratory syndrome, severe, acute)) OR (abstract:(SARS)) OR (abstract:(Respiratory syndrome, severe, acute)) OR (title:(severe acute respiratory syndrome)) OR (abstract:(severe acute respiratory syndrome))**	AND	(title:(stress OR posttraumatic) OR (abstract:(stress OR posttraumatic))	10/6/2020	1
ddc_05	**((title:(SARS)) OR (title:(Respiratory syndrome, severe, acute)) OR (abstract:(SARS)) OR (abstract:(Respiratory syndrome, severe, acute)) OR (title:(severe acute respiratory syndrome)) OR (abstract:(severe acute respiratory syndrome))**	AND	(title:(healthcare workers OR doctors OR nurses) OR (abstract:(healthcare workers OR doctors OR nurses))	10/6/2020	7
				Total	8

Search ID	Search term 1	AND/OR	Search term 2	Search date	Number of result
ndltd_01		AND		10/6/2020	62
	("SARS".ti or "SARS".ab or "非典型肺炎".ti or "非典型肺炎".ab or " 嚴重急性呼吸道症候群".ti or " 嚴重急性呼		("心理".ti or "心理".ab or "情緒".ti or "情		
ndltd_02	("SARS".ti or "SARS".ab or "非典型肺炎".ti or "非典型肺炎".ab or " 嚴重急性呼吸道症候群".ti or " 嚴重急性呼	AND	("焦慮".ti or "焦慮".ab or "憂鬱".ti or "憂	10/6/2020	12
ndltd_03	("SARS".ti or "SARS".ab or "非典型肺炎".ti or "非典型肺炎".ab or " 嚴重急性呼吸道症候群".ti or " 嚴重急性呼	AND	("壓力".ti or "壓力".ab or "睡眠".ti or "睡	10/6/2020	92
ndltd_04		AND		10/6/2020	119
	("SARS".ti or "SARS".ab or "非典型肺炎".ti or "非典型肺炎".ab or " 嚴重急性呼吸道症候群".ti or " 嚴重急性呼		("護理".ti or "護理".ab or "醫護".ti or "醫		
				Total	285

Search ID	Search term 1	AND/OR	Search term 2	Search date	Number of result
ncl_01	**嚴重急性呼吸道症候群**			10/6/2020	20
ncl_02	**(TI=SARS) [OR] (AB=SARS) [OR] (TI=非典型肺炎) [OR] (AB=非典型肺炎)**	AND	(TI=心理 OR 情緒 OR 焦慮 OR 憂鬱) OR (AB=心理 OR 情緒 OR 焦慮 OR	10/6/2020	39
ncl_03	**(TI=SARS) [OR] (AB=SARS) [OR] (TI=非典型肺炎) [OR] (AB=非典型肺炎)**	AND	(TI=壓力 OR 睡眠) OR (AB=壓力 OR 睡眠)	10/6/2020	15
ncl_04	**(TI=SARS) [OR] (AB=SARS) [OR] (TI=非典型肺炎) [OR] (AB=非典型肺炎)**	AND	(TI=護理 OR 護士 OR 醫生 OR 醫護) OR (AB=護理 OR 護士 OR 醫生 OR	10/6/2020	53
				Total	127

Search ID	Search term 1	AND/OR	Search term 2	Search date	Number of result
al_01	**((title:(SARS)) OR (title:(Respiratory syndrome, severe, acute)) OR (abstract:(SARS)) OR (abstract:(Respiratory syndrome, severe, acute)) OR (title:(severe acute respiratory syndrome)) OR (abstract:(severe acute respiratory syndrome))**	AND	(title:(mental OR psychological OR emotional)) OR (abstract:(mental OR psychological OR emotional)) OR (title:(psychiatric OR mental health OR quality of life)) OR (abstract:(psychiatric OR mental health OR quality of life))	13/6/2020	29
al_02	**((title:(SARS)) OR (title:(Respiratory syndrome, severe, acute)) OR (abstract:(SARS)) OR (abstract:(Respiratory syndrome, severe, acute)) OR (title:(severe acute respiratory syndrome)) OR (abstract:(severe acute respiratory syndrome))**	AND	(title:(mental well-being OR stigma)) OR (abstract:(mental well-being OR stigma))	13/6/2020	5
al_03	**((title:(SARS)) OR (title:(Respiratory syndrome, severe, acute)) OR (abstract:(SARS)) OR (abstract:(Respiratory syndrome, severe, acute)) OR (title:(severe acute respiratory syndrome)) OR (abstract:(severe acute respiratory syndrome))**	AND	(title:(psychosis OR depression OR anxiety) OR (abstract:(psychosis OR depression OR anxiety))	13/6/2020	3
al_04	**((title:(SARS)) OR (title:(Respiratory syndrome, severe, acute)) OR (abstract:(SARS)) OR (abstract:(Respiratory syndrome, severe, acute)) OR (title:(severe acute respiratory syndrome)) OR (abstract:(severe acute respiratory syndrome))**	AND	(title:(stress OR posttraumatic) OR (abstract:(stress OR posttraumatic))	13/6/2020	3
al_05	**((title:(SARS)) OR (title:(Respiratory syndrome, severe, acute)) OR (abstract:(SARS)) OR (abstract:(Respiratory syndrome, severe, acute)) OR (title:(severe acute respiratory syndrome)) OR (abstract:(severe acute respiratory syndrome))**	AND	(title:(healthcare workers OR doctors OR nurses) OR (abstract:(healthcare workers OR doctors OR nurses))	13/6/2020	10
				Total	50

Search ID	Search term 1	AND/OR	Search term 2	Search date	Number of result
al_01	**((title:(SARS)) OR (title:(Respiratory syndrome, severe, acute)) OR (abstract:(SARS)) OR (abstract:(Respiratory syndrome, severe, acute)) OR (title:(severe acute respiratory syndrome)) OR (abstract:(severe acute respiratory syndrome))**	AND	(title:(mental OR psychological OR emotional)) OR (abstract:(mental OR psychological OR emotional)) OR (title:(psychiatric OR mental health OR quality of life)) OR (abstract:(psychiatric OR mental health OR quality of life))	14/6/2020	2
al_02	**((title:(SARS)) OR (title:(Respiratory syndrome, severe, acute)) OR (abstract:(SARS)) OR (abstract:(Respiratory syndrome, severe, acute)) OR (title:(severe acute respiratory syndrome)) OR**	AND	(title:(mental well-being OR stigma)) OR (abstract:(mental well-being OR stigma))	14/6/2020	0
al_03	**((title:(SARS)) OR (title:(Respiratory syndrome, severe, acute)) OR (abstract:(SARS)) OR (abstract:(Respiratory syndrome, severe, acute)) OR (title:(severe acute respiratory syndrome)) OR**	AND	(title:(psychosis OR depression OR anxiety) OR (abstract:(psychosis OR depression OR anxiety))	14/6/2020	5
al_04	**((title:(SARS)) OR (title:(Respiratory syndrome, severe, acute)) OR (abstract:(SARS)) OR (abstract:(Respiratory syndrome, severe, acute)) OR (title:(severe acute respiratory syndrome)) OR (abstract:(severe acute respiratory syndrome))**	AND	(title:(stress OR posttraumatic) OR (abstract:(stress OR posttraumatic))	14/6/2020	5
al_05	**((title:(SARS)) OR (title:(Respiratory syndrome, severe, acute)) OR (abstract:(SARS)) OR (abstract:(Respiratory syndrome, severe, acute)) OR (title:(severe acute respiratory syndrome)) OR**	AND	(title:(healthcare workers OR doctors OR nurses) OR (abstract:(healthcare workers OR doctors OR nurses))	14/6/2020	7
				Total	19

Search ID	Search term 1	AND/OR	Search term 2	Search date	Number of result
lac_01	**TIOR AB=(SARS OR Severe Acute Respiratory Syndrome)**				26
				Total	26
