# Supplementary material for: History for some or lesson for all? A systematic review and meta-analysis on the immediate and long-term mental health impact of the 2002–2003 Severe Acute Respiratory Syndrome (SARS) outbreak
Source: BMC Public Health. 2021 Apr 7;21:670. doi: 10.1186/s12889-021-10701-3 (PMC8025448; doi:10.1186/s12889-021-10701-3)
Supplement: Supplementary file 2 — Additional file 2. List of excluded studies with reasons. [file 12889_2021_10701_MOESM2_ESM.pdf]

Additional file 2: List of excluded studies with reasons

Study Number	Study Name	First Author	Year	Journal	Reason for exclusion	Reason code
264	A Longitudinal Research on the Citizens' Subjective Well-being in a Coastal Province	Xing, ZJ	2005	Psychological Science	Primary data not SARS related	1
530	Impact of SARS on avian influenza preparedness in healthcare workers	Tam, DK	2007	Infection	Primary data not SARS related	1
1043	Prevalence and factors associated with social avoidance of recovered SARS patients in the Hong Kong general population	Lau, JT	2006	Health Education Research	Wrong patient population	1
1087	Quarantine: A concept to preparedness	Sibyl, S	2014	Research Journal of Pharmaceutical, Biologica	Primary data not SARS related	1
1117	Psychological approaches to the relationship between happiness and public policy in P.R. China	Shi, K	2006	Happiness and public policy	Primary data not SARS related	1
1340	Fear and stigma: the epidemic within the SARS outbreak	Person, B	2004	Emerg Infect Dis	Wrong patient population	1
1402	The awareness of late-life depression in clinical practice	Ou, WT	2017	Journal of Internal Medicine of Taiwan	Wrong setting	1
1790	Psychological status under stress in soldiers at different posts and of different service age	Li, JF	2005	Chinese Journal of Clinical Rehabilitation	Wrong patient population	1
1830	Coping with the threat of severe acute respiratory syndrome: Role of threat appraisals and coping responses in health behaviors	Lee-Baggley, D	2004	Asian Journal of Social Psychology	Beyond 5 defined regions	1
1945	The psychological burden of bioterrorism	Hall, MJ	2004	Journal of Aggression, Maltreatment and Trau	Primary data not SARS related	1
2015	Outcomes up to 5 years after severe, acute respiratory failure	Garland, A	2004	Chest	Primary data not SARS related	1
2899	One-year outcomes in survivors of the acute respiratory distress syndrome	Herridge, MS	2003	New England Journal of Medicine	Not about SARS	1
3035	Well-being in informal caregivers of survivors of acute respiratory distress syndrome	Cameron, JI	2006	Critical Care Medicine	Wrong patient population	1
619	The SARS-associated stigma of SARS victims in the post-SARS era of Hong Kong	Siu, JY	2008	Qual Health Res	Qualitative	2
36	Validation of Impact of Events Scale in nurses under threat of contagion by severe acute respiratory syndrome	Chen, CS	2005	Psychiatry and Clinical Neurosciences	validation of scale	2
131	Comparison of anxious level of medical students from different resources during severe acute respiratory syndrome epidemic period	Zuo, Q	2005	Chinese Journal of Clinical Rehabilitation	Lack clear link between morbidities and SARS	2
160	Reasons for a high score on compulsory symptom in psychological survey among college students	Zheng, LJ	2005	Chinese Journal of Clinical Rehabilitation	Lack clear link between morbidities and SARS	2
196	The impact of epidemic outbreak: the case of severe acute respiratory syndrome (SARS) and suicide among older adults in Hong Kong	Yip, PS	2010	Crisis	qualitative	2
252	Psychological Stress of Nurses in SARS Wards	Liu, XH	2003	Chinese Mental Health Journal	Lack clear link between morbidities and SARS	2
324	The cycle fear: A qualitative study of SARS and its impacts on kindergarten parents one year after the outbreak	Wong, WCW	2007	Hong Kong Practitioner	qualitative	2
477	Psychosocial Aspects in Three Universities during SARS Epidemic in Beijing	Huang, YQ	2003	Chinese Mental Health Journal	Lack clear link between morbidities and SARS	2
479	Effect of the emergency of severe acute respiratory syndrome on the psychological health in college students	Yuan, X	2004	Chinese Journal of Clinical Rehabilitation	Lack clear link between morbidities and SARS	2
496	Mental survey in female students from a medical college of Guangxi during the epidemic period of severe acute respiratory syndrome	Tang, QS	2005	Chinese Journal of Clinical Rehabilitation	Lack clear link between morbidities and SARS	2
627	Coping with future epidemics: Tai chi practice as an overcoming strategy used by survivors of severe acute respiratory syndrome (SARS) in post-SARS Hong Kong	Siu, JY	2016	Health Expect	qualitative	2
640	Psychosocial and coping responses within the community health care setting towards a national outbreak of an infectious disease	Sim, K	2010	Journal of Psychosomatic Research	qualitative	2
757	Short communication: Evaluation of the emotional status of patients on a waiting list for thoracic surgery during the outbreak of Severe Acute Respiratory Syndrome (SARS)	Wan, IYP	2004	Stress and Health: Journal of the Internationa	Lack clear link between morbidities and SARS	2
857	An exploratory study of nurses suffering from severe acute respiratory syndrome (SARS)	Mok, E	2005	Int J Nurs Pract	qualitative	2
1076	Severe acute respiratory syndrome-related psychiatric and posttraumatic morbidities and coping responses in medical staff within a primary health care setting in Singapore	Sim, K	2004	The Journal of Clinical Psychiatry	Wrong study design	2
1091	Trait-State Anxiety and Attributions to Health States in a Stressful Setting	Wang, SZ	2004	Chinese Mental Health Journal	Lack clear link between morbidities and SARS	2
1099	Surviving a life-threatening crisis: Taiwan s nurse leaders reflections and difficulties fighting the sars epidemic	Shih, FJ	2009	Journal of Clinical Nursing	Wrong study design	2
1102	Dying and caring on the edge: Taiwan's surviving nurses' reflections on taking care of patients with severe acute respiratory syndrome	Shih, FJ	2007	Appl Nurs Res	Wrong study design	2
1114	Post-traumatic stress disorder checklist following SARS	Shi, TY	2005	Chinese Journal of Clinical Rehabilitation	validation of PTSD instruments from conveni	2
1230	The psychosocial effects of being quarantined following exposure to SARS: a qualitative study of Toronto health care workers	Robertson, E	2004	Can J Psychiatry	Wrong study design	2
1250	Relationship between mental status during severe acute respiratory syndrome and type of temperament in students of medical college	Ren, ZM	2005	Chinese Journal of Clinical Rehabilitation	Lack clear link between morbidities and SARS	2
1276	The impact of severe acute respiratory syndrome on medical house staff: a qualitative study	Rambaldini, G	2005	J Gen Intern Med	Wrong study design	2
1435	If schools are closed, who will watch our kids? Family caregiving and other sources of role conflict among nurses during large-scale outbreaks	O'Sullivan, TL	2009	Prehosp Disaster Med	Wrong study design	2
1538	Bowlby and Robertson revisited: the impact of isolation on hospitalized children during SARS	Koller, DF	2006	J Dev Behav Pediatr	Wrong study design	2
1539	When family-centered care is challenged by infectious disease: pediatric health care delivery during the SARS outbreaks	Koller, DF	2006	Qual Health Res	Wrong study design	2
1583	The psychological impact of SARS on health care providers	Khee, KS	2004	Critical Care and Shock	Wrong study design	2
1678	Correlation of self-defensive styles of isolated population and their cognition to risk with mental health	Hu, SF	2005	Chinese Journal of Clinical Rehabilitation	Lack clear link between morbidities and SARS	2
1891	People's Attitudes and Emotions under Epidemic of SARS	Li, H	2003	Chinese Mental Health Journal	Wrong study design	2
1894	The SARS crisis: reflections of Hong Kong nurses	Holroyd, E	2008	Int Nurs Rev	Wrong study design	2
1921	The Socio-Psychological Effects of Killing Masked Civet in the Prevention of SARS	He, YQ	2005	Chinese Mental Health Journal	Wrong intervention	2
2037	Relationship of Mental Health to Coping Strategies Associated with SARS' Stress in College Students	Xu, FM	2004	Chinese Journal of Clinical Psychology	Wrong study design	2
2043	Mental stress and crisis intervention in the patients with SARS and the people related	Cong, Z	2003	Journal of Peking University. Health sciences	Wrong study design	2
2223	Ethical dilemmas in caring for patients with SARS	Chiang, HH	2006	Hu li za zhi The journal of nursing	Wrong study design	2
2489	New mothers' experiences of social disruption and isolation during the severe acute respiratory syndrome outbreak in Hong Kong	Dodgson, JE	2010	Nursing & Health Sciences	Wrong study design	2
2733	感染SARS醫事人員之內心感受	謝佑珊	2004	Unpublished thesis	Wrong study design	2
2736	以公共衛生觀點探討護理人員經驗SARS的復原歷程	紀滋兒	2004	Unpublished thesis	Wrong study design	2
2743	醫務社會工作者面對嚴重急性呼吸道症候群(SARS)疫情壓力因應之研究--以臺大醫院為例	左祖順	2004	Unpublished thesis	Wrong study design	2
2869	照顧SARS病患護理人員的壓力及其因應行為之初探	潘雪幸	2003	臺灣腎臟護理學會雜誌	Wrong study design	2
2934	The impact of the SARS outbreak on nurses in Hong Kong: six month after	Chung, CK	2004	HKU Theses Online (HKUTO)	Wrong study design	2
2935	Taking care of pediatric SARS patient in isolation ward: a phenomenological view	Cheung, MY	2004	HKU Theses Online (HKUTO)	Wrong study design	2
2936	The impact of SARS on elderly people in Hong Kong	Lau, MMC	2004	HKU Theses Online (HKUTO)	Wrong study design	2
2965	SARS: pregnant women's fears and perceptions	Ng, J	2004	Br. J. Midwifery	qualitative	2
2970	Psychosocial impact of SARS [2]	Tsang, HWH	2004	Emerging Infectious Diseases	Wrong study design	2
2981	Survey on SARS Related Difficulties in Work and Social Lives in Hong Kong		2003	Equal Opportunities Commission	Wrong study design	2
3033	A report of 4 cases of severe acute respiratory syndrome patients with suicide tendency	Du, L	2003	Di'er Junyi Daxue Xuebao	Wrong study design	2
46	Nurses' perceptions of severe acute respiratory syndrome: Relationship between commitment and intention to leave nursing	Chang, CS	2006	Journal of Advanced Nursing	Wrong outcomes	3

56	The impact of work-related risk on nurses during the SARS outbreak in Hong Kong	Chan, SS	2005	Family & Community Health	about nonspecific stress only	3
205	Age differences in coping and emotional responses toward SARS: A longitudinal study of Hong Kong Chinese	Yeung, DYL	2007	Aging & Mental Health	Lack clear psycho/mental elements	3
320	How to provide an effective primary health care in fighting against severe acute respiratory syndrome: the experiences of two cities	Wong, WC	2007	Am J Infect Control	Lack clear psycho/mental elements	3
322	How did general practitioners protect themselves, their family, and staff during the SARS epidemic in Hong Kong?	Wong, WC	2004	J Epidemiol Community Health	Lack structured measures	3
328	The psychological impact of severe acute respiratory syndrome outbreak on healthcare workers in emergency departments and how they cope	Wong, TW	2005	Eur J Emerg Med	about nonspecific stress only	3
354	Psychological responses to the SARS outbreak in healthcare students in Hong Kong	Wong, JGWS	2004	Medical Teacher	about nonspecific stress only	3
405	Caring for those who care: the role of the occupational health nurse in disasters	Tomczyk, D	2008	Aaohn j	Lack clear psycho/mental elements	3
406	SARS: coping with the impact at a community hospital	Tolomiczenko, G	2005	Journal of Advanced Nursing	Lack structured measures	3
427	SARS--a perspective from a school of nursing in Hong Kong	Thompson, DR	2004	J Clin Nurs	Lack clear psycho/mental elements	3
443	The relational activation of resilience model: How leadership activates resilience in an organizational crisis	Teo, WL	2017	Journal of Contingencies and Crisis Managem	Lack clear psycho/mental elements	3
461	Controlled study of posttraumatic stress disorder among patients with severe acute respiratory syndrome and first-line hospital staffs as well as public in prevalent areas	Zhang, KR	2005	Chinese Journal of Clinical Rehabilitation	Lack structured measures	3
622	Qigong practice among chronically ill patients during the SARS outbreak	Siu, JY	2007	J Clin Nurs	Lack clear psycho/mental elements	3
666	Bits of falling sky and global pandemics: Moral panic and Severe Acute Respiratory Syndrome (SARS)	Muzzatti, SL	2005	Illness, Crisis, & Loss	Lack clear psycho/mental elements	3
842	Experiencing SARS: perspectives of the elderly residents and health care professionals in a Hong Kong nursing home	Tse, MM	2003	Geriatr Nurs	about nonspecific stress only	3
866	Behaviour, Cognition and Emotion of the Public in Beijing towards SARS	Qian, MY	2003	Chinese Mental Health Journal	about nonspecific stress only	3
920	The immediate psychological and occupational impact of the 2003 SARS outbreak in a teaching hospital	Maunder, R	2003	Cmaj	Lack structured measures	3
954	Comparative stigma of HIV/AIDS, SARS, and tuberculosis in Hong Kong	Mak, WW	2006	Soc Sci Med	Lack clear psycho/mental elements	3
957	Social support and psychological adjustment to SARS: the mediating role of self-care self-efficacy	Mak, WW	2009	Psychol Health	Lack structured measures	3
960	A comparative study of the stigma associated with infectious diseases (SARS, AIDS, TB)	Mak, WW	2009	Hong Kong Med J	Lack clear psycho/mental elements	3
970	Relations of SARS-related stressors and coping to Chinese college students' psychological adjustment during the 2003 Beijing SARS epidemic	Main, A	2011	J Couns Psychol	Lack clear psycho/mental elements	3
1029	Tachycardia amongst subjects recovering from severe acute respiratory syndrome (SARS)	Lau, ST	2005	Int J Cardiol	Wrong outcomes	3
1070	The SARS (Severe Acute Respiratory Syndrome) pandemic in Hong Kong: effects on the subjective wellbeing of elderly and younger people	Lau, AL	2008	Aging Ment Health	Lack clear psycho/mental elements	3
1107	Factors predicting nurses' consideration of leaving their job during the SARS outbreak	Shiao, JS	2007	Nurs Ethics	Wrong outcomes	3
1115	Post-traumatic stress disorder and related factors following the severe acute respiratory syndrome	Shi, T. Y.	2005	Chinese Journal of Clinical Rehabilitation	did not specify cutoff criteria for diagnosis o	3
1116	Quality of life after discharge in patients with severe acute respiratory syndrome	Shi, L	2005	Chinese Journal of Clinical Rehabilitation	Lack clear psycho/mental elements	3
1118	Rationality of 17 cities' public perception of SARS and predictive model of psychological behavior	Shi, K	2003	Chinese Science Bulletin	Lack structured measures	3
1272	Godzilla in the corridor: The Ontario SARS crisis in historical perspective	Rankin, J	2006	Intensive Crit Care Nurs	about nonspecific stress only	3
1287	Crisis prevention and management during SARS outbreak, Singapore	Quah, SR	2004	Emerg Infect Dis	Lack clear psycho/mental elements	3
1292	Behavioural, cognitive and emotional responses to SARS: Differences between college students in Beijing and Suzhou	Qian, MY	2005	Stress and Health: Journal of the Internationa	Lack structured measures	3
1309	The impact of ERI, burnout, and caring for SARS patients on hospital nurses' self-reported compliance with infection control	Pratt, M.	2009	Canadian Journal of Infection Control	Lack clear psycho/mental elements	3
1334	Infectious respiratory disease outbreaks and pregnancy: occupational health and safety concerns of Canadian nurses	Phillips, KP	2011	Prehosp Disaster	Lack clear psycho/mental elements	3
1475	Outcome of coronavirus-associated severe acute respiratory syndrome using a standard treatment protocol	Lau, AC	2004	Respirology	Wrong outcomes	3
1481	Sharing the trauma: Guidelines for therapist self-disclosure following a catastrophic event	LaPorte, H	2010	Best Practices in Mental Health: An Internatior	Lack clear psycho/mental elements	3
1507	The lessons of SARS in Hong Kong	Lai, TS	2010	Clin Med (Lond)	Lack structured measures	3
1628	The Risk Perceptions of SARS and Socio-Psychological Behaviors of Urban People In China	Shi, K	2003	Acta Psychologica Sinica	Lack clear psycho/mental elements	3
1643	A Confirmative Research on Social Support and SARS Stress	Tone, HJ	2004	Psychological Science	Lack clear psycho/mental elements	3
1644	Long-term sequelae of SARS: physical, neuropsychiatric, and quality-of-life assessment	Hui, DSC	2009	Hong Kong Medical Journal	Lack clear psycho/mental elements	3
1665	Model of SARS Stress and its Character	Tone, HJ	2004	Acta Psychologica Sinica	Lack clear psycho/mental elements	3
1685	Confidence in controlling a SARS outbreak: experiences of public health nurses in managing home quarantine measures in Taiwan	Hsu, CC	2006	Am J Infect Control	Lack clear psycho/mental elements	3
1726	Mood state of netizens in association with their coping style and social support under the stress of severe acute respiratory syndrome	Lin, XY	2005	Chinese Journal of Clinical Rehabilitation	Lack clear psycho/mental elements	3
1728	The Mediating Effects of Coping Ways on Quality of Life of Persons Recovered from SARS	Shi, L	2004	Chinese Journal of Clinical Psychology	Lack clear psycho/mental elements	3
1736	Lessons learned from the anti-SARS quarantine experience in a hospital-based fever screening station in Taiwan	Lin, EC	2010	Am J Infect Control	Lack clear psycho/mental elements	3
1777	An investigation of response to SARS stress and mental health of nurse students during SARS prevalent period	Li, XL	2005	Chinese Journal of Clinical Rehabilitation	Lack structured measures	3
1795	Long-term sequelae of SARS in children	Li, AM	2004	Paediatr Respir Rev	Lack structured measures	3
1798	Community Reactions to the SARS Crisis in Hong Kong: Analysis of a Time-Limited Counseling Hotline	Leung, TTF	2005	Journal of Human Behavior in the Social Envir	No standardised measures	3
1799	Repositioning risk in social work education: Reflections arising from the threat of SARS to social work students in Hong Kong during their field practicum	Leung, TTF	2007	Social Work Education	Lack clear psycho/mental elements	3
1804	A tale of two cities: community psychobehavioral surveillance and related impact on outbreak control in Hong Kong and Singapore during the severe acute respiratory syndrome ep	Leung, GM	2004	Infect Control Hosp Epidemiol	Lack clear psycho/mental elements	3
1846	Facing SARS: Psychological impacts on SARS team nurses and psychiatric services in a Taiwan general hospital	Lee, SH	2005	General Hospital Psychiatry	Lack clear psycho/mental elements	3
1848	The experience of SARS-related stigma at Amoy Gardens	Lee, S	2005	Soc Sci Med	Lack clear psycho/mental elements	3
1905	Fear of severe acute respiratory syndrome (SARS) among health care workers	Ho, SM	2005	J Consult Clin Psychol	Lack clear psycho/mental elements	3
1974	The occupational and psychosocial impact of SARS on academic physicians in three affected hospitals	Grace, SL	2005	Psychosomatics	Lack clear psycho/mental elements	3
2012	Experiences and implications of social workers practicing in a pediatric hospital environment affected by SARS	Gearing, RE	2007	Health & Social Work	Lack clear psycho/mental elements	3
2022	Flexible coping responses to severe acute respiratory syndrome-related and daily life stressful events	Gan, YQ	2004	Asian Journal of Social Psychology	Lack clear psycho/mental elements	3
2060	Rapid assessment of a helpdesk service supporting severe acute respiratory syndrome patients and their relatives	Chung, JW	2004	Journal of Clinical Nursing	Lack clear psycho/mental elements	3
2061	SARS: caring for patients in Hong Kong	Chung, BP	2005	Journal of Clinical Nursing	Wrong outcomes	3
2065	Psychometric evaluation of the stress scale of caring for highly infectious disease patients among health care workers - Based on SARS	Chuang, PY	2005	Taiwan Journal of Public Health	Lack clear psycho/mental elements	3
2090	SARS and psychogeriatrics: Perspective and lessons from Hong Kong	Chiu, HFK	2003	International Journal of Geriatric Psychiatry	Wrong outcomes	3
2156	The original report of mental assistance hotline for SARS in Beijing	Zhong, J	2003	Chinese Mental Health Journal	Lack clear psycho/mental elements	3
2169	The Effects of Different Coping Strategy on People's Behavior Habits Under the Stressor of SARS	Wang, JP	2004	Chinese Journal of Clinical Psychology	Lack clear psycho/mental elements	3
2171	Optimism across cultures: In response to the severe acute respiratory syndrome outbreak	Ji, LJ	2004	Asian Journal of Social Psychology	Lack clear psycho/mental elements	3

2194	How infectious disease outbreaks affect community-based primary care physicians: comparing the SARS and H1N1 epidemics	Jaakkimainen, RL	2014	Can Fam Physician	Lack clear psycho/mental elements	3
2224	Self-state of nurses in caring for SARS survivors	Chiang, HH	2007	Nursing Ethics	Wrong outcomes	3
2235	Institutional trust as a determinant of anxiety during the SARS crisis in Hong Kong	Cheung, CK	2008	Social Work in Public Health	Lack structured measures	3
2253	Perception of benefits and costs during SARS outbreak: An 18-month prospective study	Cheng, C	2006	Journal of Consulting and Clinical Psychology	Lack structured mental health morbidities me	3
2265	The impact of the SARS outbreak on an urban emergency department in Taiwan	Chen, WK	2005	Medical Care	Lack structured measures	3
2268	Post-SARS knowledge sharing and professional commitment in the nursing profession	Chen, SL	2008	Journal of Clinical Nursing	Lack clear psycho/mental elements	3
2494	Factors influencing compliance with quarantine in Toronto during the 2003 SARS outbreak	DiGiovanni, C	2004	Biosecurity and Bioterrorism-Biodefense Stra	Wrong outcomes	3
2500	The Singaporean response to the SARS outbreak: knowledge sufficiency versus public trust	Deurenberg-Yap	2005	Health Promotion International	Wrong outcomes	3
2508	Perceived threat, risk perception, and efficacy beliefs related to SARS and other (emerging) infectious diseases: Results of an international survey	deZwart, O	2009	International Journal of Behavioral Medicine	Wrong outcomes	3
2684	Comparison of the mental health level during severe acute respiratory syndrome between 1041 students of five colleges in Inner Mongolia and national norms	Bai, YX	2005	Chinese Journal of Clinical Rehabilitation	Lack clear psycho/mental elements	3
2707	Strategies adopted and lessons learnt during the severe acute respiratory syndrome crisis in Singapore	Tan, BH	2005	Rev Med Virol	Wrong outcomes	3
2738	Job Stress and Coping in Emergency Room Nurses Confronting Severe Acute Respiratory Syndrome Crisis	Yu, WC	2004	Chung Shan Medical Journal	about nonspecific stress only	3
2773	The Singaporean response to the SARS outbreak: Knowledge sufficiency versus public trust Risk perception and impact of severe acute respiratory syndrome (SARS) on work and p	Deurenberg-Yap	2005	Health Promotion International	Lack clear psycho/mental elements	3
2807	SARS治疗一线医护人员心理健康状况调查及危机干预模式构建的研究	汪萍	2005	unpublished thesis	Lack structured measures	3
2885	SARS期間臺灣觀光旅館之公司政策、SARS知識與員工心理焦慮之關係研究	許順旺	2006	餐旅暨家政學刊	Lack structured measures	3
2895	Clinical mental state of sufferer of SARS intervention mode and curative effect valuation	Wang, ZQ	2003	Zhongguo Xinli Weisheng Zazhi	Lack structured measures	3
2900	The Impact of the SARS Epidemic on the Utilization of Medical Services: SARS and the Fear of SARS	Chang, HJ	2004	American Journal of Public Health	Lack clear psycho/mental elements	3
2907	SARS疫情對大專生心理影響與預防認知之研究--以美和技術學院為例	莊德豐	2007	美和技術學院學報	Lack structured measures	3
2912	急診病患接受SARS防疫隔離看診的心理衝擊與照護滿意度	彭逸祺	2004	臺灣急診醫學會醫誌	Lack clear psycho/mental elements	3
3010	The mental health status of medical workers in SARS wards and outpatient departments in Beijing	Yang, X	2004	Chin J Health Educ	Lack structured measures	3
3021	The public's response to severe acute respiratory syndrome in Toronto and the United States	Blendon, RJ	2004	Clinical Infectious Diseases	Lack clear psycho/mental elements	3
275	Relation between social support and mental health of patients with severe acute respiratory syndrome	Xiao, R	2004	Chinese Journal of Clinical Rehabilitation	Validation of the instrument cannot be foun	3
2719	防治传染性非典型肺炎过程中保健医务人员心理状态调查	张晓红	2003	中国临床康复	doubtful design and validity of the questionr	3
3006	Behaviour, Cognition and Emotion of the Public in Beijing towards SARS. [Behaviour, Cognition and Emotion of the Public in Beijing towards SARS.]	Qian, MY	2003	Chinese Mental Health Journal	doubtful design and validity of the questionr	3
3012	Study of the effects of SARS on the mental health of the medical staff of a fever clinic in a military hospital [Chinese]	Liu, J	2004	Nanfang J Nurs	doubtful design and validity of the questionr	3
2940	Longitudinal changes in community psycho-behavioural responses and impact on outbreak control during severe acute respiratory syndrome(SARS) epidemic in Hong Kong	Fong, HC	2004	HKU Theses Online (HKUTO)	likely used a subset of data from Leung et al.	4
274	Investigation on psychological condition of patients with severe acute respiratory syndrome	Xiao, R	2004	Chinese Journal of Clinical Rehabilitation	Redundant content in another full paper	4
301	Posttraumatic stress, anxiety, and depression in survivors of severe acute respiratory syndrome (SARS)	Wu, KK	2005	J Trauma Stress	Redundant content in another full paper	4
369	Mental health impact of severe acute respiratory syndrome: a prospective study	Wing, YK	2012	Hong Kong Med J	Redundant content in another full paper	4
698	Chronic fatigue, pain, depression and disordered sleep in chronic HIV and post SARS patients	Moldofsky, H	2012	26th Annual Meeting of the Associated Profe	Redundant content in another full paper	4
855	Fatigue, muscular symptoms and disordered sleep in chronic post SARS	Moldofsky, H	2005	Sleep	Redundant content in another full paper	4
1006	The Mental Health of Hospital Workers Dealing with Severe Acute Respiratory Syndrome	Lu, YC	2006	Psychotherapy and Psychosomatics	data overlapped with other included studies	4
1040	SARS-related perceptions in Hong Kong	Lau, JTF	2005	Emerging Infectious Diseases	Redundant content in another full paper	4
1125	The effects of disease severity, use of corticosteroids and social factors on neuropsychiatric complaints in severe acute respiratory syndrome (SARS) patients at acute and convalesc	Sheng, B	2005	Eur Psychiatry	Data overlapped with other included studies	4
1652	The long-term impact of severe acute respiratory syndrome (SARS) on pulmonary function, exercise capacity, and quality of life in a cohort of survivors	Hui, DS	2005	Chest	Redundant content in another full paper	4
1653	Impact of severe acute respiratory syndrome (SARS) on pulmonary function, functional capacity and quality of life in a cohort of survivors	Hui, DS	2005	Thorax	Redundant content in another full paper	4
1809	The impact of community psychological responses on outbreak control for severe acute respiratory syndrome in Hong Kong	Leung, GM	2003	J Epidemiol Community Health	redundant content in another full paper	4
2745	嚴重急性呼吸道症候群醫療照護者之心理健康	呂宜靜	2006	高雄醫學大學行為科學研究所學位論文	Redundant content in another full paper	4
3011	The psychological impact of an infectious disease outbreak: Lessons from SARS in Beijing	Wu, P	2007	American Public Health Association 135th An	Redundant content in another full paper	4
1333	Coping responses of emergency physicians and nurses to the 2003 severe acute respiratory syndrome outbreak	Phua, D	2005	Acad Emerg Med	same data and result as #435 without additic	4
938	The relevance of psychosocial variables and working conditions in predicting nurses' coping strategies during the SARS crisis: an online questionnaire survey	Marjanovic, Z	2007	Int J Nurs Stud	data overlapped with other included studies	4
278	Outcomes of SARS survivors in China: not only physical and psychiatric co-morbidities	Xiang, YT	2014	East Asian Arch Psychiatry	Not an original research or review	5
572	SARS control and psychological effects of quarantine, Toronto, Canada - In response	Styra, R	2005	Emerging Infectious Diseases	Not an original research or review	5
800	Combating stigma and fear: Applying psychosocial lessons learned from the HIV epidemic and SARS to the current Ebola crisis	Vega, MY	2016	The psychosocial aspects of a deadly epidemic:	Not an original research or review	5
854	Psychosocial impact of SARS	Tsang, HW	2004	Emerg Infect Dis	Not an original research or review	5
898	Psychological morbidity related to the SARS outbreak in Hong Kong	McAlonan, GM	2005	Psychol Med	Not an original research or review	5
909	Applying the lessons of SARS to pandemic influenza: an evidence-based approach to mitigating the stress experienced by healthcare workers	Maunder, R	2008	Can J Public Health	Not an original research or review	5
922	The experience of the 2003 SARS outbreak as a traumatic stress among frontline healthcare workers in Toronto: lessons learned	Maunder, R	2004	Philos Trans R Soc Lond B Biol Sc	Not an original research or review	5
923	Stress, coping and lessons learned from the SARS outbreak	Maunder, R	2003	Hosp Q	Not an original research or review	5
1073	The psychological impact of SARS: a matter of heart and mind	Sim, K	2004	Cmaj	Not an original research or review	5
1210	Severe acute respiratory syndrome: "Unmasking our emotional vulnerability as healthcare providers"	Ruppert-Garcia,	2004	Critical Care and Shock	Not an original research or review	5
1968	Professional Efficacy and Social Support in Nurses During the SARS Crisis in Canada and China	Greenglass, ER	2011	New Directions in Organizational Psychology a	Not an original research or review	5
2016	Psychological Impact on SARS Survivors: Critical Review of the English Language Literature	Gardner, PJ	2015	Canadian Psychology-Psychologie Canadienne	Not an original research or review	5
2249	Psychological intervention with sufferers from severe acute respiratory syndrome (SARS): Lessons learnt from empirical findings	Cheng, SK	2005	Clinical Psychology & Psychotherapy	Not an original research or review	5
2485	Community mobilisation and empowerment for combating a pandemic	Dong, WZ	2010	Journal of Epidemiology and Community Hea	Not an original research or review	5
2512	Work-related critical incidents in hospital-based health care providers and the risk of post-traumatic stress symptoms, anxiety, and depression: A meta-analysis	de Boer, J	2011	Social Science & Medicine	Not an original research or review	5
2716	The yellow peril revisited: the impact of SARS on Chinese and Southeast Asian Communities	Leung, C	2008	Resources for Feminist Research 2008 Spring	Not an original research or review	5
2752	灾难时期的社区心理干预——中国SARS疫情发生后的社区精神卫生问题	黄悦勤	2003	中华全科医师杂志	Not an original research or review	5
2801	突发事件时实施群体心理危机干预的效果研究	金宁宁	2005	unpublished thesis	Not an original research or review	5
2991	Affirming flames: Stress management for nurses under SARS outbreak	Chan, C	2003	The Hong Kong Nursing Journal	Not an original research or review	5

3015	To be paranoid is the standard? Panic responses to SARS outbreak in the Hong Kong Special Administrative Region	Cheng, C	2004	Asian Perspectiv	Not an original research or review	5
3026	Severe acute respiratory syndrome (SARS): A brief review with exploration of the outcomes, prognostic factors and sequelae	Chan JW	2005	Current Respiratory Medicine Reviews	Not an original research or review	5
3042	Mental stress and crisis intervention in the patients with SARS and the people related	Cong, Z	2003	Journal of Peking University. Health sciences	Not an original research or review	5
469	Psychological crisis intervention model in Xiaotanshan Hospital of PLA	Wang, ZQ	2003	Chinese Mental Health Journal	confusing data	6
559	Psychological analysis for the medical staff suffering from severe acute respiratory syndrome	Sun, H	7609	Chinese Journal of Clinical Rehabilitation	Lack key information	6
869	Predictive factors of psychological disorder development during recovery following SARS outbreak	Mihashi, M.	2009	Health Psychol	a mix of patients and non-patients, not defin	6
1789	Observation of curative effect of mental crisis comprehensive in 669 patients with severe acute respiratory syndrome	Li, JM	2006	Chinese Journal of Clinical Rehabilitation	Inconsistent data	6
2295	Does social capital matter when medical professionals encounter the SARS crisis in a hospital setting	Chang, KH	2006	Health Care Management Review	outcome measure questionable validity	6
2451	Investigation to psychological status of the students in the medical observing area of severe acute respiratory syndrome in Sichuan University	Fang, L	2004	Chinese Journal of Clinical Rehabilitation	Inconsistent figures	6
2721	SARS患者与抗SARS医务人员心理健康对比分析	王发强	2003	中国心理卫生杂志	No specification on when the measurement w	6
2746	護理人員對SARS衝擊之身心健康:三年預後研究	凌美貝	2007	義守大學管理研究所碩士班	doubtful validity of data	6
2815	SARS患者PTSD相关因素分析及追踪研究	孙燕	2005	山西医科大学 硕士研究生毕业论文	Mixing SARS and suspected SARS patient in on	6
265	SCL-90 Results of Medical Staffs treating SARS	Xin, Y	2003	Chinese Mental Health Journal	data inconsistency	6
266	The 'Typhoon Eye Effect': Determinants of distress during the SARS epidemic	Xie, XF	2011	Journal of Risk Research	data of mental health measures cannot be ext	6
2602	The psychological impact of quarantine and how to reduce it: rapid review of the evidence	Brook, S	2020	Lancet	Not primary study	5
2603	A Systematic, Thematic Review of Social and Occupational Factors Associated With Psychological Outcomes in Healthcare Employees During an Infectious Disease Outbreak	Brook, S	2018	Journal of Occupational and Environmental M	Not primary study	5
107	Survey of stress reactions among health care workers involved with the SARS outbreak	Bai, YX	2004	Psychiatric Services	Non-validated instrument	3

Coding scheme of reasons of exclusion

Wrong study population	1
Wrong study design	2
Lack standardised measures of mental health morbidities	3
Data overlapped with other included studies	4
Not primary research	5
Data not extractible	6
