# Supplementary material for: History for some or lesson for all? A systematic review and meta-analysis on the immediate and long-term mental health impact of the 2002–2003 Severe Acute Respiratory Syndrome (SARS) outbreak
Source: BMC Public Health. 2021 Apr 7;21:670. doi: 10.1186/s12889-021-10701-3 (PMC8025448; doi:10.1186/s12889-021-10701-3)
Supplement: Supplementary file 3 — Additional file 3. Supplementary figures. [file 12889_2021_10701_MOESM3_ESM.docx]

Additional file 3: Supplementary figures.


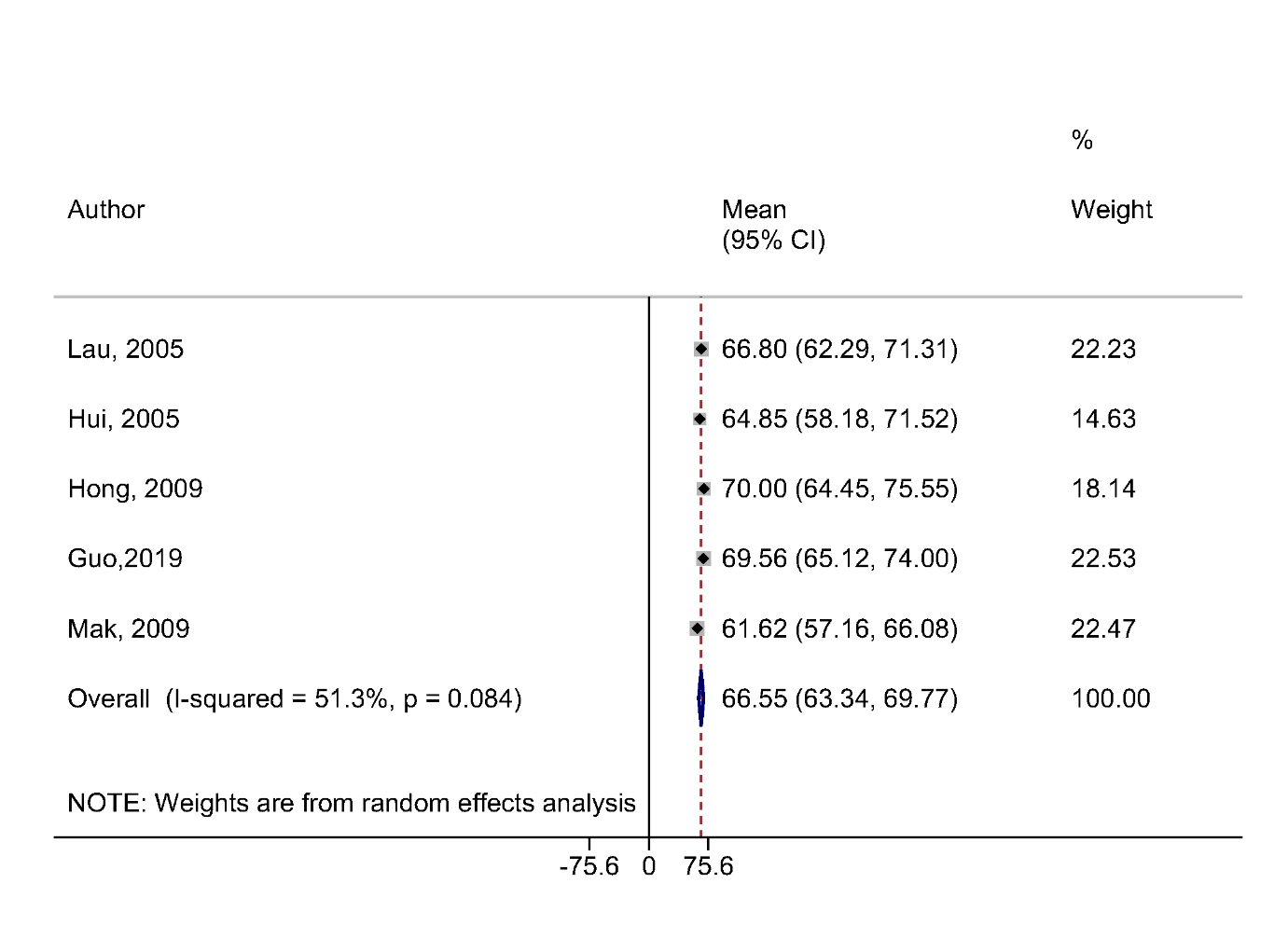
Supplementary Figure 1. Forest plot of pooled estimate of mean health-related QOL using SF-36 (mental health) among SARS patients


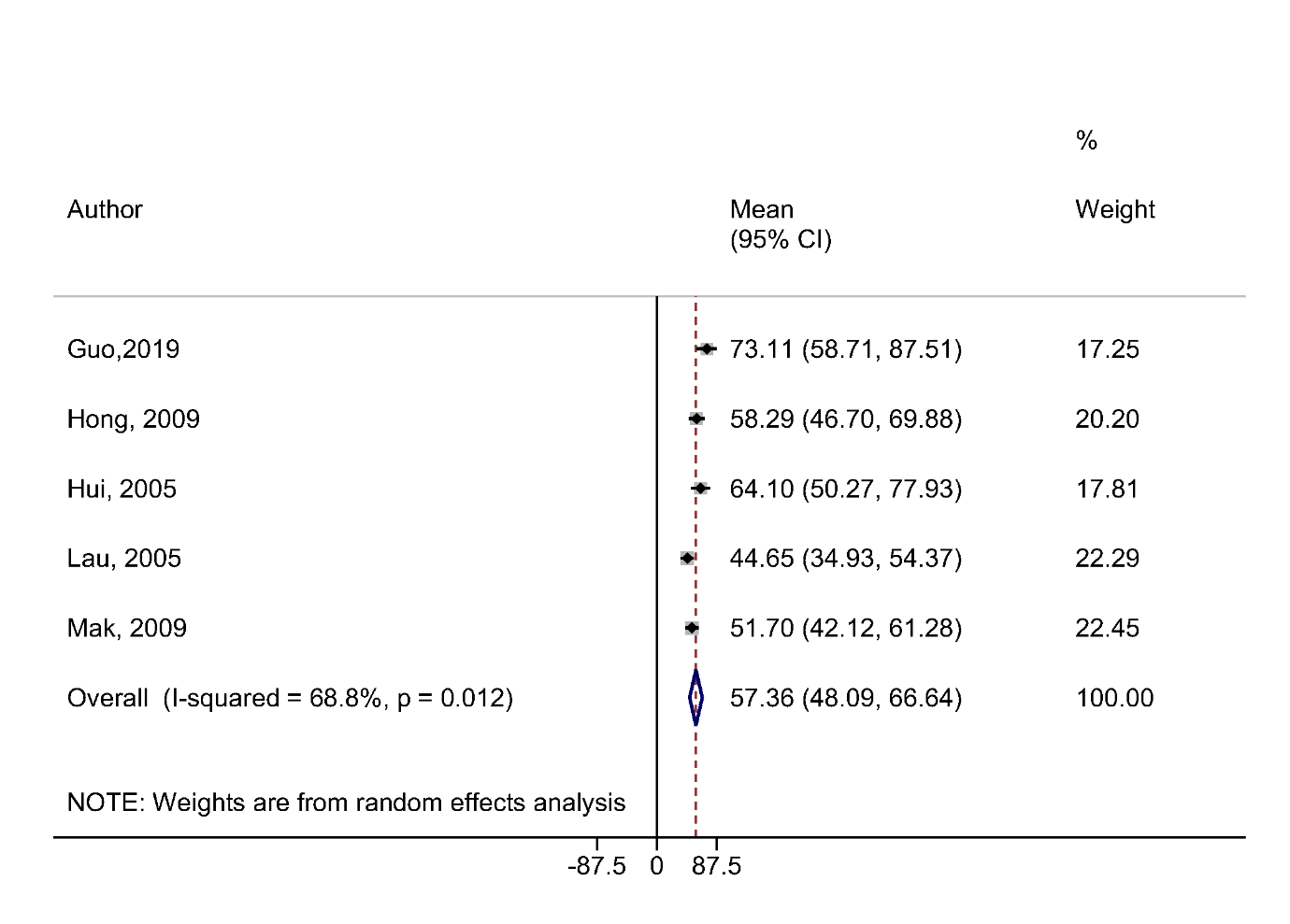
Supplementary Figure 2. Forest plot of pooled estimate of mean health-related QOL using SF-36 (role emotional) among SARS patients


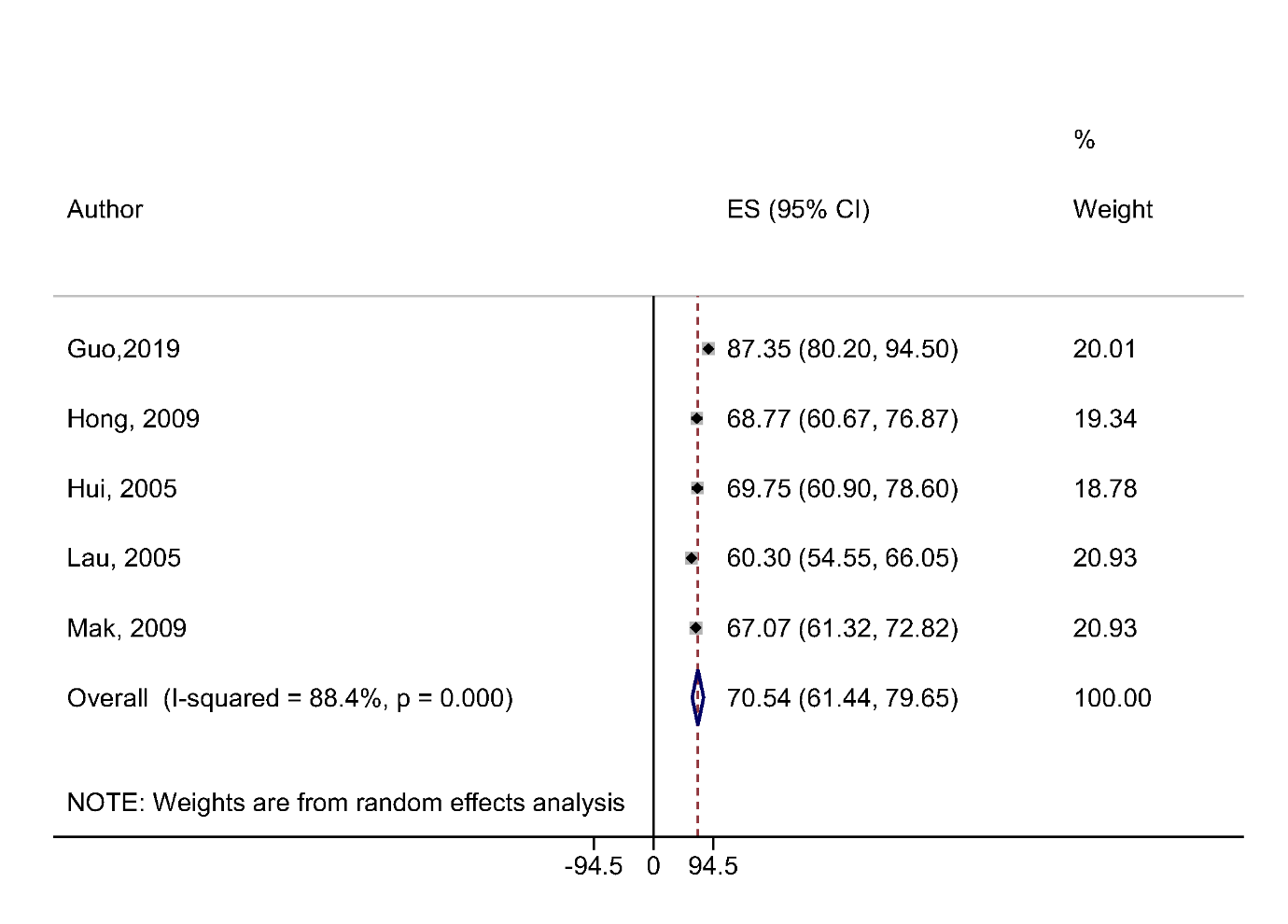
Supplementary Figure 3. Forest plot of pooled estimate of mean health-related QOL using SF-36 (social functioning) among SARS patients


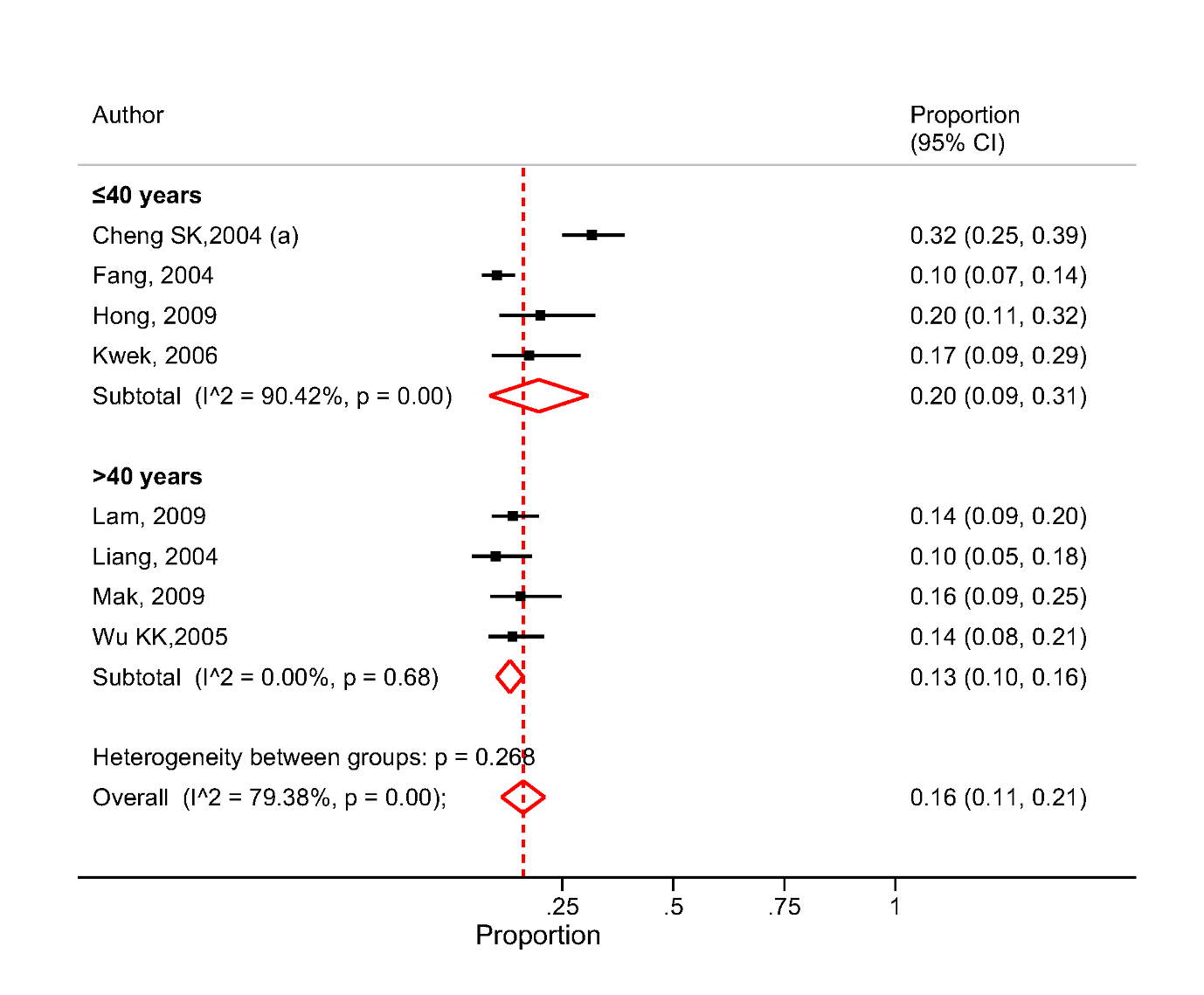
Supplementary Figure 4. Prevalence of anxiety among SARS patients stratified by age


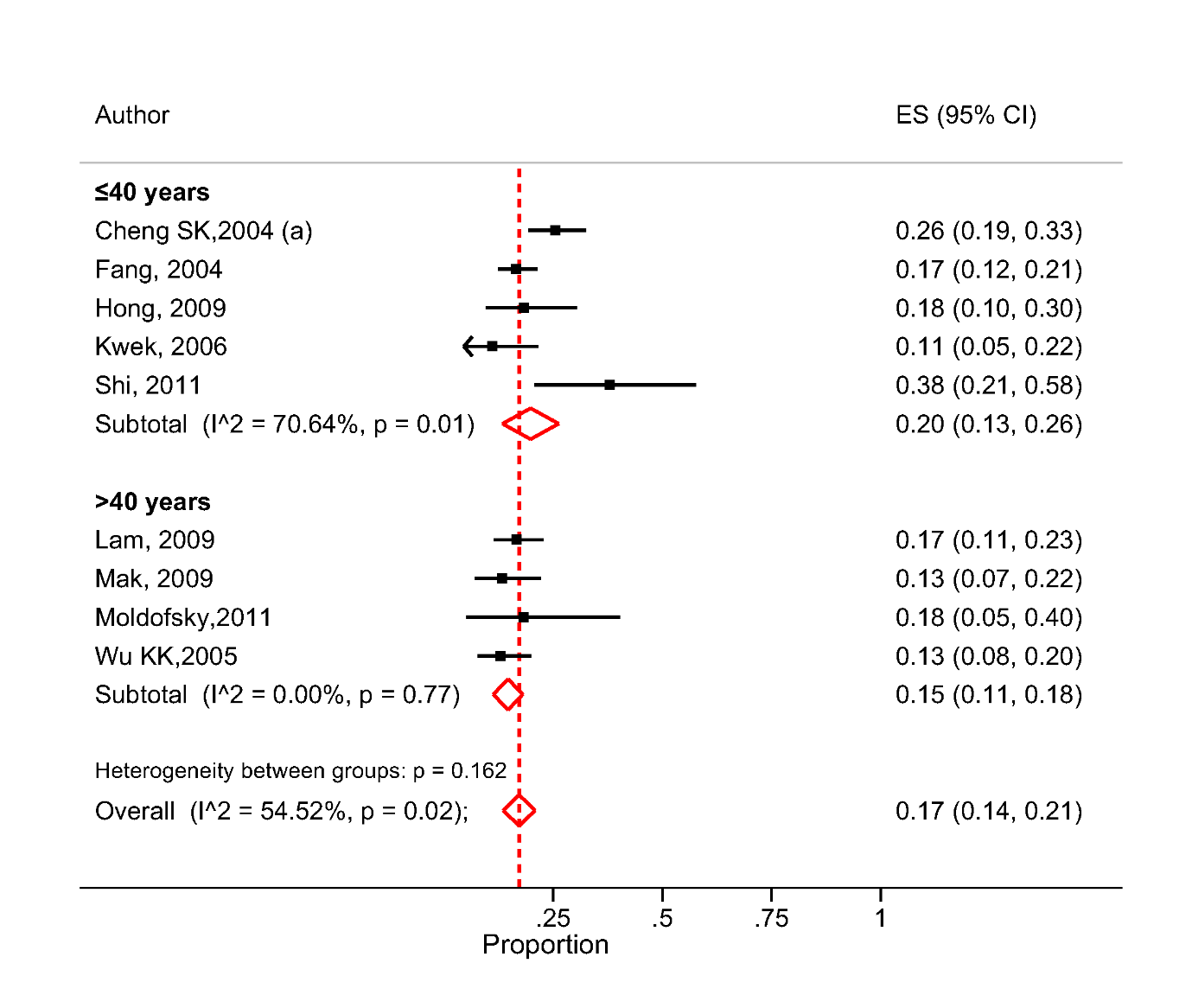
Supplementary Figure 5. Prevalence of depression among SARS patients stratified by age


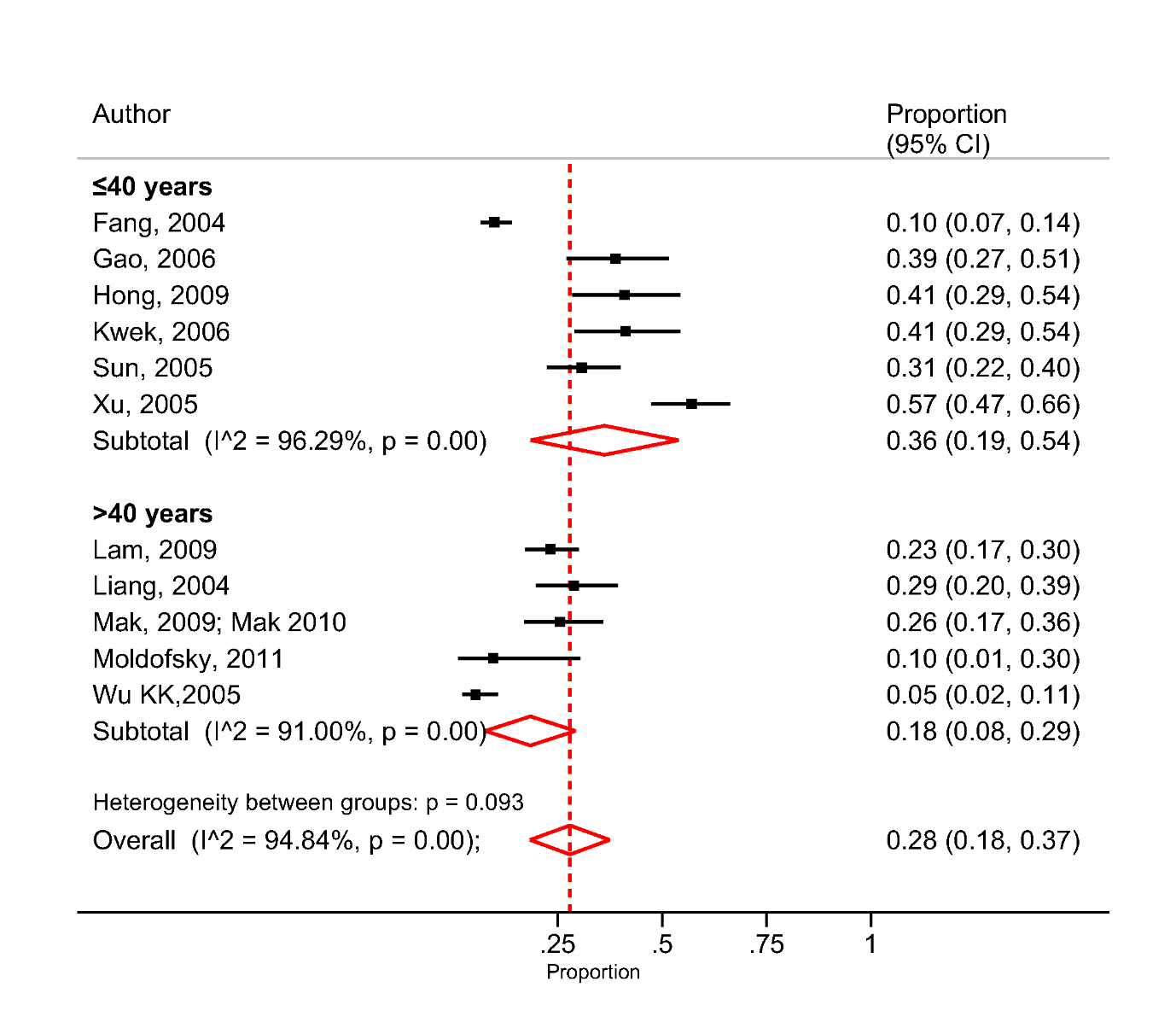
Supplementary Figure 6. Prevalence of PTSD among SARS patients stratified by age


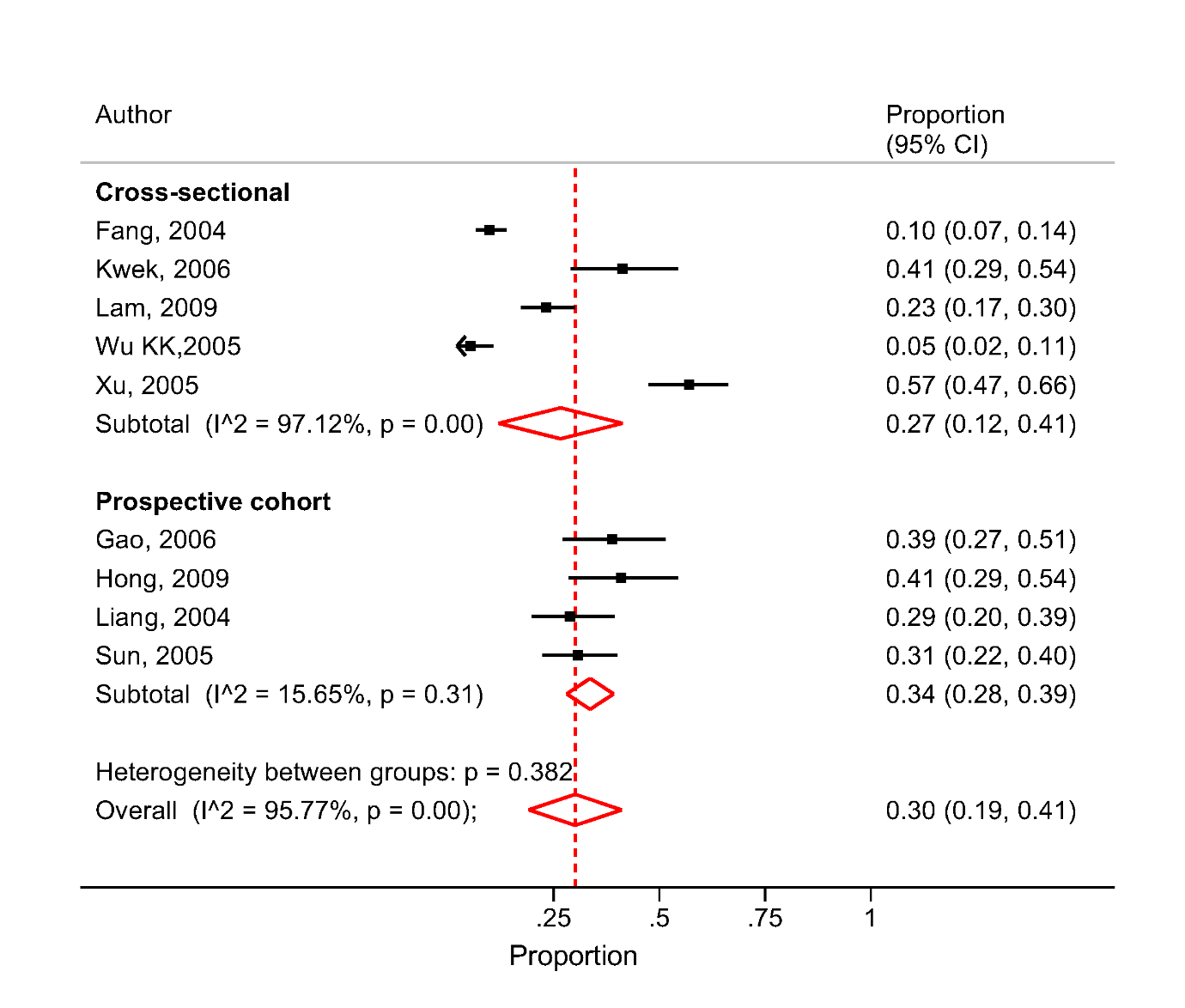
Supplementary Figure 7. Prevalence of PTSD among SARS patients stratified by study design


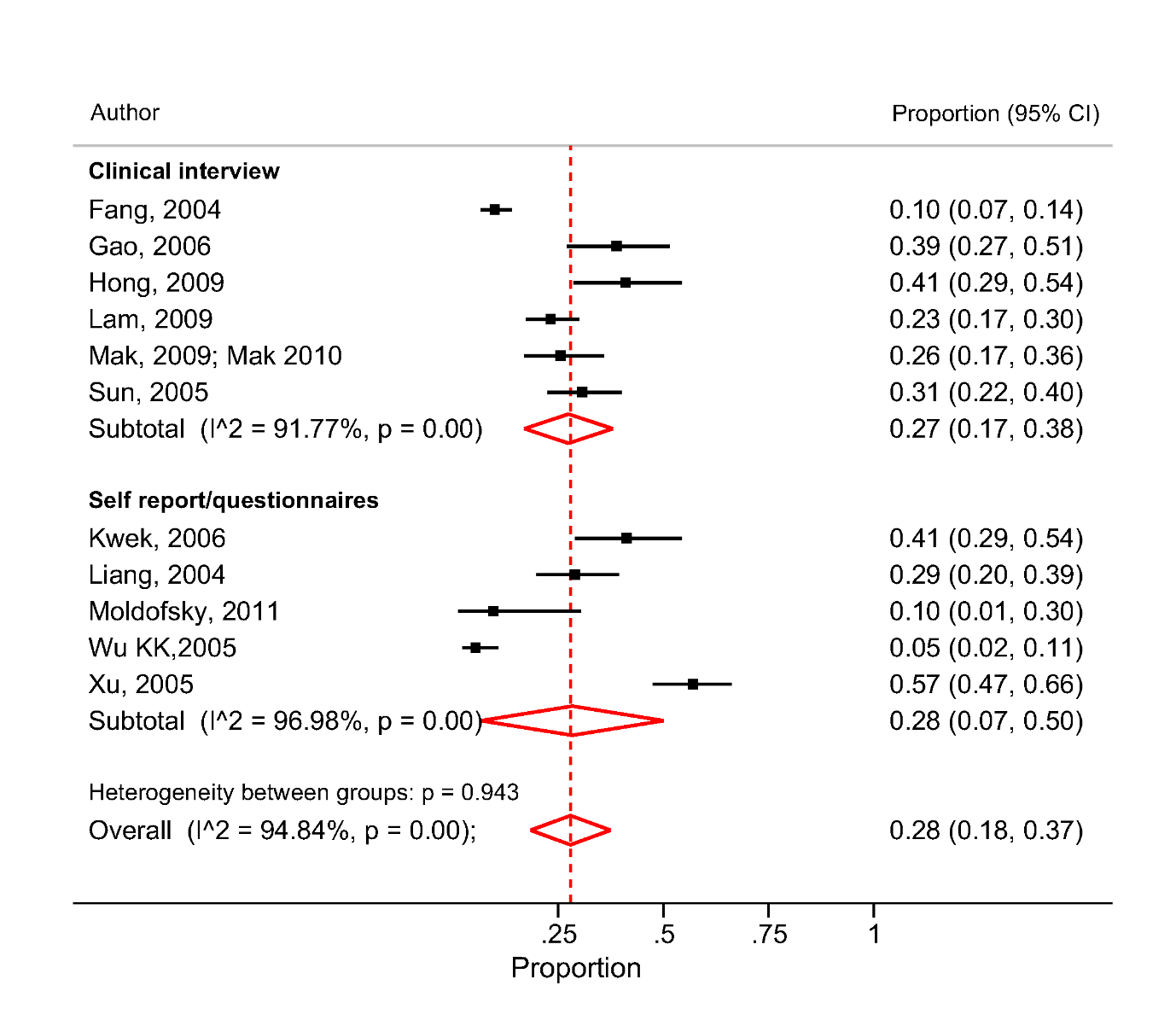
Supplementary Figure 8. Prevalence of PTSD among SARS patients -comparison between clinical interviews and self-report/questionnaire diagnosis


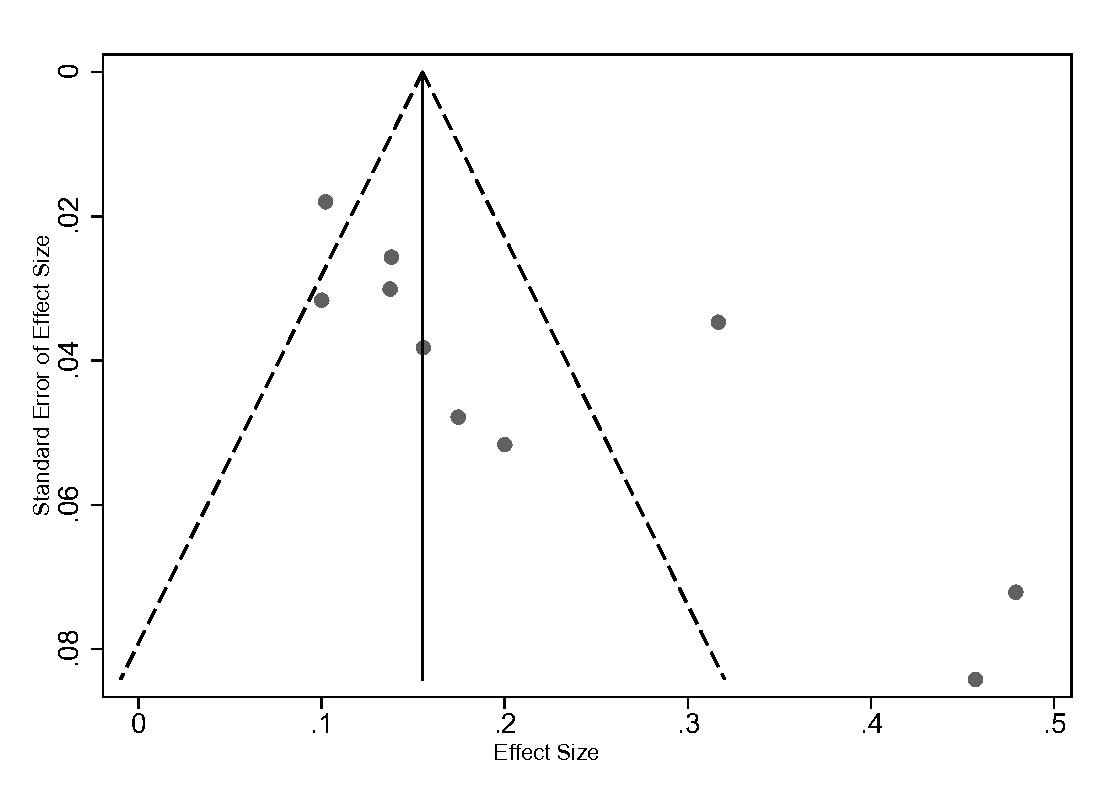
Supplementary Figure 9. Funnel plot with pseudo 95% confidence limits for studies included in prevalence estimate of anxiety among SARS patients


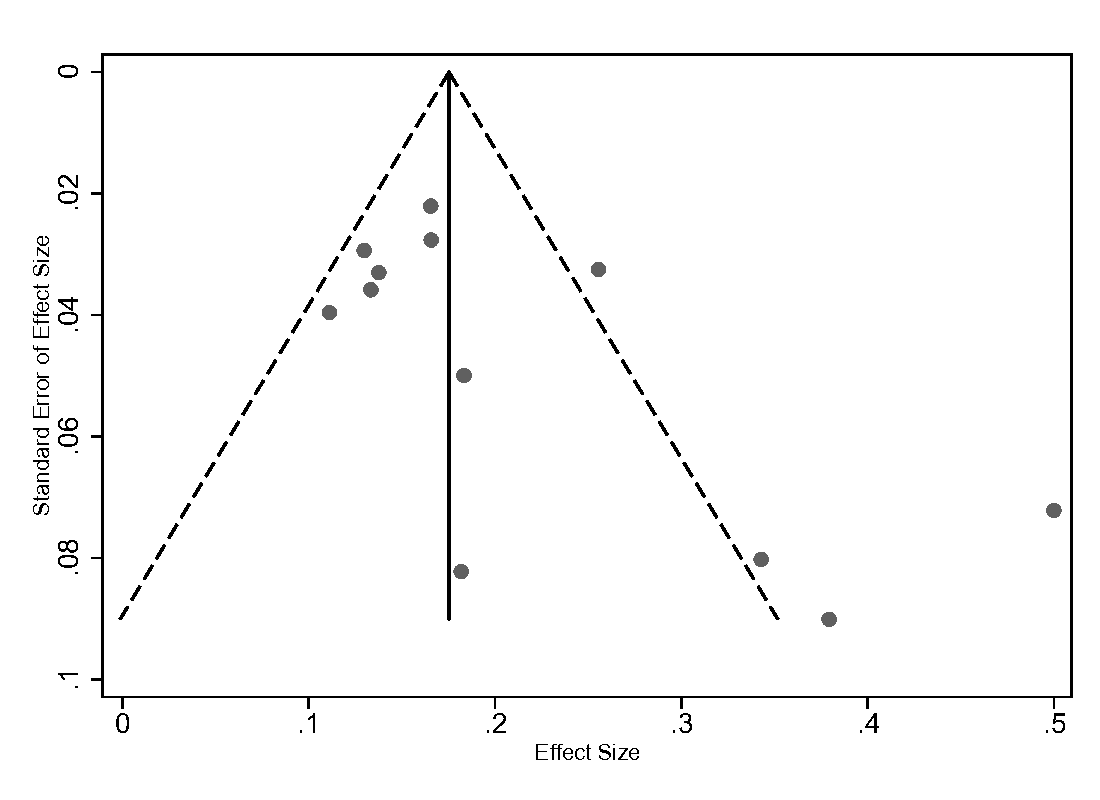
Supplementary Figure 10. Funnel plot with pseudo 95% confidence limits for studies included in prevalence estimate of depression among SARS patients


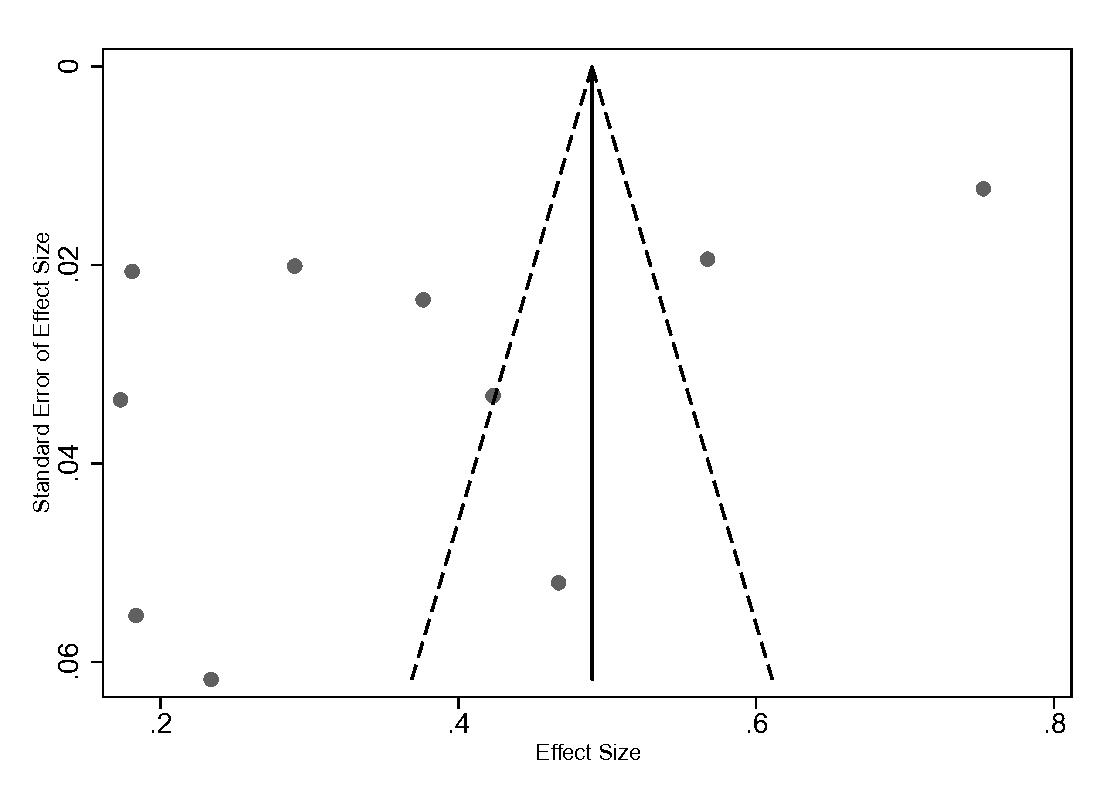
Supplementary Figure 11. Funnel plot with pseudo 95% confidence limits for studies included in prevalence estimate of general psychological distress among health care professionals


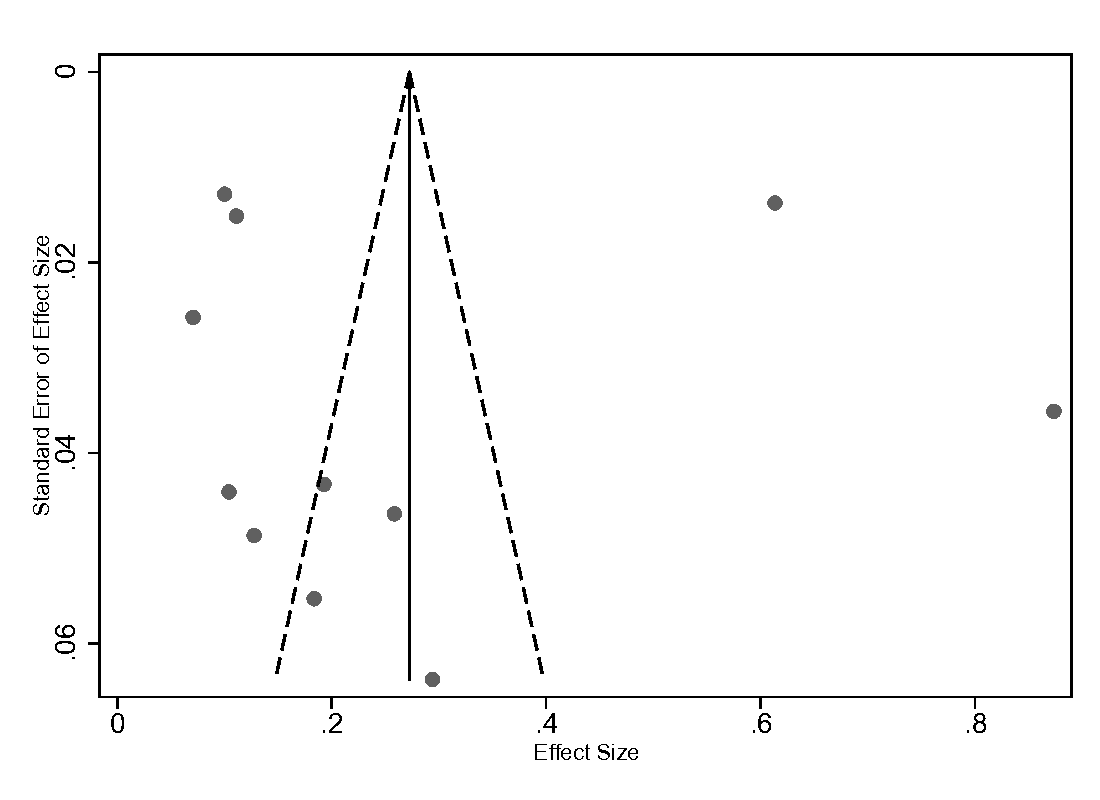
Supplementary Figure 12. Funnel plot with pseudo 95% confidence limits for studies included in prevalence estimate of PTSD among health care professionals


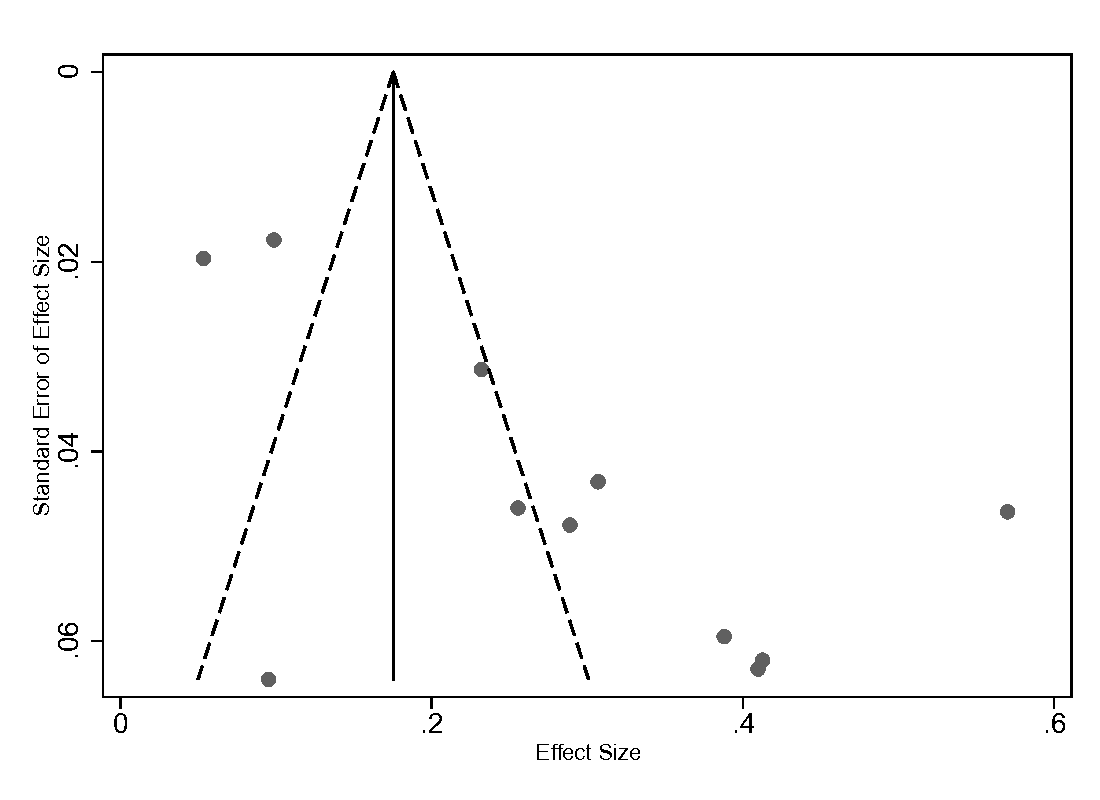
Supplementary Figure 13. Funnel plot with pseudo 95% confidence limits for studies included in prevalence estimate of PTSD among SARS patients
